# Molecular Pap Smear: Validation of HPV Genotype and Host Methylation Profiles of *ADCY8*, *CDH8*, and *ZNF582* as a Predictor of Cervical Cytopathology

**DOI:** 10.3389/fmicb.2020.595902

**Published:** 2020-10-15

**Authors:** Jane Shen-Gunther, Qingqing Xia, Winfred Stacey, Heisy B. Asusta

**Affiliations:** ^1^Gynecologic Oncology & Clinical Investigation, Department of Clinical Investigation, Brooke Army Medical Center, Fort Sam Houston, TX, United States; ^2^Department of Molecular Medicine, Cancer Therapy and Research Center, University of Texas Health Science Center at San Antonio, San Antonio, TX, United States; ^3^Department of Clinical Investigation, Brooke Army Medical Center, Fort Sam Houston, TX, United States; ^4^Department of Obstetrics and Gynecology, Brooke Army Medical Center, Fort Sam Houston, TX, United States

**Keywords:** carcinogenesis, DNA methylation, epigenetic modification, evolution, host-pathogen interactions, human papillomavirus infection, pap smear, pyrosequencing

## Abstract

Primary high-risk Human Papillomavirus (hrHPV) screening has recently become an accepted standalone or co-test with conventional cytology. Unfortunately, hrHPV singularly lacks specificity for cytopathological grade. However, mechanisms and markers of evolving virus-host interactions at the epigenome level may be harnessed as a better predictor of carcinogenesis. This study aimed to validate and expand the clinical performance of a multiparametric biomarker panel, referred to as the “Molecular Pap smear” based, on HPV genotype and *ADCY8*, *CDH8* and *ZNF582* CpG-methylation as a predictive classifier of cervical cytology. This prospective, cross-sectional study used an independent cohort of residual liquid-based cytology for HPV genotyping and epigenetic analysis. Extracted DNA underwent parallel PCR using 3 primer sets for HPV DNA amplification. HPV-infected samples were genotyped by Sanger sequencing. Promoter methylation levels of 3 tumor suppressor genes were quantified by bisulfite-pyrosequencing of genomic DNA on the newest high-resolution PyroMark Q48 platform. Logistic model performance was compared, and model parameters were used to predict and classify binary cytological outcomes. A total of 883 samples were analyzed. HPV DNA positivity correlated with worsening grade: 125/237 (53%) NILM; 136/235 (58%) ASCUS; 222/229 (97%) LSIL; and 157/182 (86%) HSIL samples. The proportion of carcinogenic HPV-types in PCR-positive sequenceable samples correlated with worsening grade: NILM 34/98 (35%); ASCUS 50/113 (44%); LSIL 92/214 (43%); HSIL 129/152 (85%). Additionally, *ADCY8*, *CDH8*, and *ZNF582* methylation levels increased in direct correlation with worsening grade. Overall, the multi-marker modeling parameters predicted binarized cytological outcomes better than HPV-type alone with significantly higher area under the receiver operator curve (AUC)s, respectively: NILM vs. > NILM (AUC 0.728 vs. 0.709); NILM/ASCUS vs. LSIL/HSIL (AUC 0.805 vs. 0.776); and <HSIL vs. HSIL (AUC 0.830 vs. 0.761). Our expanded findings validated the multivariable prediction model developed for cytological classification. The sequencing-based “Molecular Pap smear” outperformed HPV-type alone in predicting four grades of cervical cytology. Additional host epigenetic markers that evolved with disease progression decidedly contributed to the overall classification accuracy.

## Introduction

May 13, 2019 marked the 136th birthday of Dr. George Papanicolaou who was honored globally with an iconic image of his pioneering work on cervical cancer screening and the Papanicolaou (Pap) smear (Papanicolaou and Traut, 1941; Maxouris, 2019). Armed with a microscope, glass slides, cellular stains, and his wife, Andromachi (his life-long control subject), Papanicolaou changed the world of cancer prevention and founded a new scientific discipline called cytopathology (Carmichael and Cameron, 1973). Although 80 years have passed since the invention of the Pap smear, impenetrable economic and social barriers have prevented this life-saving test from reaching impoverished regions of the world (Canfell et al., 2020).

On World Cancer Day (4 February 2020), the World Health Organization (WHO) announced that a 60% increase in cancer cases worldwide is projected over the next two decades (World Health Organization [WHO] News Release, 2020). Currently, a staggering 18 million new cancer cases are diagnosed globally each year of which 13% (2.2 million) are caused by infectious agents (Wild et al., 2020). Human papillomavirus (HPV) ranks second only to Helicobacter pylori as the primary infectious cause of cancer. Annually, HPV is responsible for 570,000 new cervical and 120,000 other anogenital and oropharyngeal cancer cases (Wild et al., 2020). Low- and middle-income countries continue to carry the highest cancer burden and will incur the greatest increase in cancer incidence and mortality in the years to come due to inadequate resources for cancer prevention and early detection (World Health Organization [WHO], 2014, 2020).

To circumvent the formidable economic and infrastructural requirements associated with cytology-based screening programs and limitations of commercial HPV diagnostics, we developed a molecular diagnostic test called “Molecular Pap smear” which is based on HPV genotyping and quantitative DNA methylation. Fundamentally, the test harnesses the evolutionary characteristics of the pathogen, the host and infected host-tissue throughout carcinogenesis for use as biomarkers (Bosch et al., 2013; Chen et al., 2018). Our prior investigation had shown a loss of HPV genotypic diversity and gain of clonal dominance by carcinogenic genotypes in high-grade versus low-grade cytology (Shen-Gunther et al., 2017). Additionally, distinct patterns of loci-specific promoter hypermethylation were discovered and were consistent with the underlying mechanism of HPV E6 and E7 oncoprotein induced DNA methyltransferase activity and ensuing gene silencing (Durzynska et al., 2017). Our initial study which analyzed ∼300 cervical cytology samples indicated HPV genotype and host promoter methylation may perform well as a molecular classifier of cervical cytopathology. Furthermore, this work showed that the positive correlation between 1) HPV carcinogenicity, 2) *ADCY8, CDH8*, and *ZNF582* promoter hypermethylation as well as 3) grade of cervical pathology were quantifiable and distinctive.

In this study, we aimed to validate and expand the clinical performance of our multiparametric biomarker panel with an independent sample set inclusive of four cytological categories [negative for intraepithelial lesion or malignancy (NILM), atypical squamous cells of undetermined significance (ASC-US), low-grade squamous intraepithelial lesion (LSIL), and high-grade squamous intraepithelial lesion (HSIL)]. Secondarily, we sought to validate quantitative CpG-methylation by pyrosequencing (PSQ) on the newest high-resolution PyroMark 48 Autoprep platform, which to our knowledge has not yet been reported in the literature in contrast to the preceding models, Q24 and Q96 (Johannessen et al., 2018). The results of this study will aid in the translation of our current discoveries based on virus-host evolutionary characteristics of HPV-induced carcinogenesis into a screening test that is more accurate, affordable, and widely available to improve global health (Turajlic et al., 2015; Beerenwinkel et al., 2016).

## Materials and Methods

### Subjects and Samples

This prospective cross-sectional study was conducted after approval by the Institutional Review Board of Brooke Army Medical Center (BAMC), Texas. Cervical specimens were collected from adult women ≥ 18 years of age undergoing cervical cytology screening. Cervical specimens with low cellularity (cell pellet volume < 200 uL) were excluded from analysis. Liquid-based cytology collected for clinical testing at the Department of Pathology of BAMC was consecutively procured after completion of analysis for cytological diagnosis. Samples were stored at room temperature until weekly batch DNA extraction. Demographic data were abstracted from the electronic health record (AHLTA) of the Department of Defense (DoD) and code-linked to each specimen. Four categories of samples were collected until target accrual numbers totaling 883 samples were met [NILM (*n* = 237), ASCUS (*n* = 235), LSIL (*n* = 229), and HSIL (*n* = 182)].

### Laboratory Schema

**FIGURE 1:**
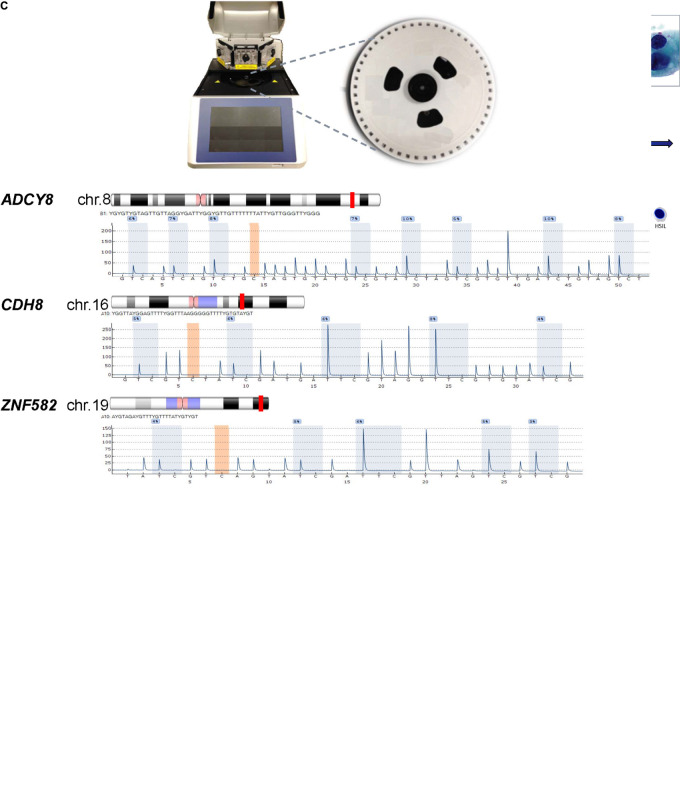
Protocol schema and representative images of four cervical cytological grades used in the study. **(A)** Sample collection, DNA extraction, HPV genotyping by Sanger sequencing and genomic CpG profiling of loci-specific promoters by PSQ. Sequencing results are used for statistical modeling, prediction and classification. **(B)** Four categories of liquid-based cervical cytology: NILM, ASC-US, LSIL, and HSIL with cytomorphologic features of disease progression, i.e., increased nuclear enlargement, nuclear membrane irregularity, nuclear/cytoplasmic ratio, and chromatin coarseness (ThinPrep Pap smear, 50x magnification). Bottom, binarized classification of 4 cytological grades used as outcomes (“0” and “1”) for logistic regression. Three distinct, sequential logit models were used for outcome prediction by molecular signatures. **(C)** PyroMark Q48 PSQ instrument and 48-well sample disk (expanded) used for DNA methylation analysis. Bottom, the PSQ CpG assays for three host genes: *ADCY8, CDH8, and ZNF582* are shown according to chromosomal locations (red line). The representative pyrograms with assay specific CpG sites (blue-gray columns) are shown with sequence specific, light-intensity peaks along the x- and y-axis, respectively. Chromosome ideograms adapted from NCBI Map Viewer (www.ncbi.nlm.nih.gov/genome/guide/human)]. ASC-US, atypical squamous cells of undetermined significance; chr, chromosome; gDNA, genomic DNA; HSIL, high-grade squamous intraepithelial lesion; LSIL, low-grade squamous intraepithelial lesion; NILM, negative for intraepithelial lesion or malignancy; PCR, polymerase chain reaction; PSQ, pyrosequencing. Photo credit (cytology): Bradie Bishop, MD.

The laboratory schema is illustrated in Figure 1A. After sample collection, cellular DNA is extracted from cervical cytology. The DNA is subjected to HPV DNA amplification, sequencing, and genotyping. For DNA methylation analysis, the genomic DNA undergoes bisulfite conversion and PSQ. The results derived from HPV genotyping and methylation quantification are analyzed for correlation with the cytological grade. Figure 1B shows representative images of the 4 categories of cervical cytology used in this study and binarization schema of cytological outcomes for logistic regression, prediction, and classification. Morphological features and differences among the cytological categories are highlighted by the relative size and distribution of organelles. The PyroMark Q48 instrument and PSQ assays are shown in Figure 1C.

### HPV DNA Amplification

Cellular DNA extraction and HPV DNA amplification were performed as described previously (Shen-Gunther et al., 2016). Briefly, individual liquid-based cytology samples (10 mL) were centrifuged to obtain the cell pellet (200–250 μL) for DNA extraction using the QIAamp DNA Mini kit in a QIAcube robotic workstation (Qiagen). The purified DNA in 150 μL of eluent was quantified by spectrophotometry using the QIAxpert (Qiagen) and stored at -20°C prior to amplification. For HPV DNA amplification, 3 consensus primer sets: (1) MY09/11, (2) FAP59/64, and (3) GP-E6-3F/GP-E7-5B/GP-E7-6B were used to amplify 2 distinct regions of the HPV L1 and E6/E7 genes for genotype identification, respectively, as illustrated in Supplementary Figure 1 (Resnick et al., 1990; Forslund et al., 1999; Sotlar et al., 2004; Shen-Gunther and Yu, 2011). Functionally, E6 and E7 codes for oncoproteins which inactivate two respective host cellular proteins p53 and RB leading to malignant transformation. L1 codes for the structural capsid protein which is essential for viral binding and entry into host tissues (Buck et al., 2013). AmpliTaq Gold 360 Master Mix (Life Technologies) and Qiagen Multiplex PCR Plus kit (Qiagen) were used with the doublet and triplet primer sets, respectively. The PCR cycling protocols for the 3 primer sets were also performed as described previously (Shen-Gunther et al., 2016). After amplification, high-resolution capillary gel electrophoresis using the QIAxcel (Qiagen) was performed to detect HPV DNA amplicons for follow-on DNA sequencing.

### HPV DNA Sequencing, Genotyping, and Phylogenetic Analysis

Sanger sequencing of the amplicons (∼200 ng DNA/sample) was performed by using sequencing primers MY11, FAP59, and GP-E6-3F at Eurofins Genomics (Louisville, KY). Sequence quality was assessed using the Sequence Scanner 2.0 (appliedbiosystems.com), where a “high quality” Trace Score (TS) (average base call quality value as measured by phred quality score) was defined as ≥20 and a QV20 + value (total number of bases in the sequence with TS ≥ 20) as ≥100. Quality sequences were filter selected for entry into the Basic Local Alignment Search Tool (BLAST^®^) and queried against HPV sequences in GenBank^®^ under Virus Taxonomy ID#151340 (Shen-Gunther and Yu, 2011) using the CLC Genomics Workbench v20.0.4 (Redwood City, CA). The HPV genotype was based on the most homologous and significant result with a minimum Expected Value (*E*-value) < 10E^–50^. For each sample, if the identified HPV genotype differed among the primer-specific amplicons, the genotype with the lowest *E*-value took precedence. Furthermore, if the *E*-values were equal between sequenced amplicons (e.g., *E*-value = 0), the genotype was assigned in descending order of precedence by the primer-specific amplicon: E6/E7, MY09/11, and FAP59/64. The rationale for this ranking was based on the clinical significance of HPV E6/E7 over L1 gene function in respect to carcinogenic potential. The proportions of samples in which HPV was detected according to genotype and genotype-specific carcinogenic potential within each cytological category were compared.

To explore the evolutionary relationship of all HPV genotypes identified in the clinical samples, a representative phylogenetic tree was inferred using the Neighbor-Joining method (Saitou and Nei, 1987) after concatenating the aligned, E6 (∼477 bp), E7 (∼297 bp), and L1 (∼1,576 bp) reference coding sequences from Papillomavirus Episteme^1^ by MUSCLE (Edgar, 2004). The evolutionary distances were computed using the Maximum Composite Likelihood method (Tamura et al., 2004). Codon positions included were 1st + 2nd + 3rd + Non-coding. Positions containing gaps or missing data were eliminated. Bootstrap analysis using 1,000 replicates was performed to evaluate the reliability of the inferred tree (Felsenstein, 1985). Evolutionary analyses were conducted in MEGA X (Kumar et al., 2018).

### DNA Methylation Quantification

For DNA methylation profiling of cervical cytology, extracted genomic DNA (≥ 20 ng/uL) was bisulfite-converted to convert unmethylated cytosine residues to uracil using the EpiTect Fast 96 Bisulfite Conversion kit (Qiagen) per the manufacturer’s instructions. Loci-specific PCR amplification of the bisulfite-converted gDNA (10–20 ng) was performed using 3 primer pairs targeting *ADCY8*, *CDH8*, or *ZNF582* combined with Pyromark PCR Master Mix (Qiagen) per the manufacturer’s instructions. The PSQ assays including primer sequences were performed as described previously (Shen-Gunther et al., 2016). The PCR reaction (25 uL volume) and cycling protocol were performed per manufacturer’s instructions and are described as follows: activation [95°C × 15 min]; 45 cycles of 3-step cycling [94°C × 30 s, 56°C × 30 s, 72°C × 30 s]; and final extension [72°C × 10 min].

The biotinylated PCR product was analyzed using high-resolution capillary gel electrophoresis (QIAxcel) for expected size in base pairs and adequacy of DNA concentration (>1 ng/uL) prior to PSQ (Supplementary Figure 2). The PCR product (10 uL) and magnetic beads (3 uL) were pipetted into the PyroMark Q48 disk wells and loaded on the Q48 instrument (Qiagen) for PSQ (Figure 1C). Post-run results for CpG methylation quantification were analyzed using the Q48 Autoprep software 2.4.2 on CpG analysis mode and visualized as sequence-specific pyrograms (Figure 1C). The individual CpG-methylation levels (%) of each sample were joined with HPV status to construct the multivariable logistic models below.

### Definitions, Variable Coding, and Logistic Modeling

The WHO International Agency for Research on Cancer (IARC) Working Group classifies HPV carcinogenic potential into three primary categories (International Agency for Research on Cancer [IARC], 2012) (1) carcinogenic (HPV types 16, 18, 31, 33, 35, 39, 45, 51, 52, 56, 58, 59, and 68) (2) possibly carcinogenic (HPV types 26, 30, 34, 53, 66, 67, 69, 70, 73, 82, 85, and 97) and (3) not classifiable/probably not carcinogenic (HPV types 6, 11, and all others) (Schiffman et al., 2009; Bernard et al., 2010).

To compare the prevalence of HPV genotypes grouped by carcinogenicity among the 4 cytological categories, the HPV genotype found in each sample was coded on an ordinal scale: HPV undetected (0), not classifiable/not carcinogenic (1), possibly carcinogenic (2), carcinogenic (3), and highly carcinogenic, (4). Cytology was also coded on an ordinal scale, NILM (0), ASC-US (1), LSIL (2), and HSIL (3), to determine the correlation between HPV carcinogenicity and cytological grade. For CpG-methylation levels (%), a percentile definition was used. The 95th percentile value for each CpG derived from NILM cytology (HPV-negative) was used as the cut-off for normal methylation (coded as 0); >95th percentile was deemed hypermethylated (coded as 1).

Preceding logistic regression modeling, missing data from the explanatory variables were handled by chained multiple imputation, which fills in missing values of multiple variables iteratively by using chained specifications of prediction equations based on the distribution of each variable (StataCorp, 2019). Multivariable logistic regression (Long and Freese, 2014) was performed to determine the association between the methylation level of each CpG locus of 3 genes (*ADCY8, CDH8*, and *ZNF582*) and a binarized cytological outcome of interest. Outcome Model 1 aimed to distinguish normal (NILM) from abnormal (ASC-US/LSIL/HSIL) cytology, Model 2 distinguished NILM/ASC-US from LSIL/HSIL cytology, and Model 3 distinguished HSIL cytology from all others (NILM/ASC-US/LSIL). The covariates (CpG site(s) selected from each gene) that had the highest association with the response variable (*p*-value < 0.05) were entered in a 2nd multivariable logistic regression jointly with HPV carcinogenicity to select the explanatory variables most predictive of the cytological outcome. The 2nd model equation is as follows:

$$\begin{array}{c} \mathrm{Logistic}\mathrm{model}:\mathrm{Probability}\mathrm{of}\mathrm{outcome}=P\left(Y=1\right)\\ =1/1+e^{\wedge }\left[-\left(b_{0}+b_{1}X_{1}+\cdots +b_{4}X_{4}\right)\right] \end{array}$$

Multiple explanatory variables: X_1_,…, X_4_.
X_1_ = HPV carcinogenicity (coded as ordinal data as described above).
X_2_ = *ADCY8* CpG-site *i* methylation (0, 1).
X_3_ = *CDH8* CpG-site *i* methylation (0, 1).
X_4_ = *ZNF582* CpG-site *i* methylation (0, 1).
Binarized Model 1 Outcome (Y) coding: NILM (0), ASC-US/LSIL/HSIL (1).
Binarized Model 2 Outcome (Y) coding: NILM/ASC-US (0), LSIL/HSIL (1).
Binarized Model 3 Outcome (Y) coding: NILM/ASC-US/LSIL (0), HSIL (1).

For the final regression models, post estimation receiver operating characteristic (ROC) curves were constructed and predictions at specified values were computed. After estimating the classification threshold or cut-off for each model by using Youden’s index (maximum sum of sensitivity and specificity), diagnostic performance characteristics were determined (Youden, 1950). Of note, a cut-off may be adjusted for greater or lesser sensitivity (true positives) while trading off 1-specificity (false positives) based on relative importance of the parameters and purpose of a clinical test. The discriminatory performance between multivariable and univariable (HPV carcinogenicity) models was compared using respective areas under the ROC curve. Pairwise comparisons of predicted probabilities between models were performed with the chi-square test.

### Statistical Analysis

This study was designed to have a 90% power to detect a 10% difference in DNA methylation (%) between successive categories of cytology. From the literature, locus-specific promoter methylation levels (%) for NILM, LSIL/HSIL and cervical cancer have ranged from 0–5%, 15–30%, and 30–60%, respectively (Lai et al., 2008; Wentzensen et al., 2009; Siegel et al., 2015). To detect a 10% difference in methylation levels using a one-sided test set at α = 0.05 and β = 0.10 with an allocation ratio of 1, a total accrual target of *N* = 306 and *n* = 153 per group was required. The quota sampling strategy assured adequate representation from each cytological grade. Additional samples were collected to compensate for potential sample inadequacy and laboratory errors.

Data were summarized using means (95% CI), medians (IQR), and proportions. For hypothesis testing, Wilcoxon rank sum and Kruskal-Wallis tests were used for non-parametric, numerical, or ordinal data. Categorical data were compared using the chi-square test. Correlation between ordinal variables was determined by Spearman’s rho. *p*-values < 0.05 were considered statistically significant. Statistical analyses were performed using STATA/IC 16.0 (StataCorp LP).

## Results

### HPV Type-Specific Carcinogenicity Correlates With Cytological Grade

A total of 883 residual cytology samples were collected between September 2015 and March 2017. Clinical and cytological characteristics are summarized in Table 1. The corresponding subjects were composed predominantly of Caucasians (38%) with a median age of 30 years (IQR, 25–37). The cytological specimens were stratified proportionately among the 4 grades except for HSIL with fewer samples (chi-square *p* < 0.05): NILM 237/883 (27%); ASC-US 235/883 (26%); LSIL 229/883 (26%), and HSIL 181/883 (21%). Sample #503 was classified as HSIL/SCC and the 66-year old patient was subsequently diagnosed of Stage IIA invasive SCC. This sample thus served as the positive control for this study. The median concentrations of extracted DNA among the 4 cytological categories were adequate and statistically equivalent (range, 91.6–119.2 ng/uL) (Kruskal-Wallis test, *p* = 0.519) (Table 1).

**TABLE 1:** Clinical and cytological characteristics of the study population.

Characteristics	N	(%)^h^
**Clinical**		
**Age^a^**		
Median (IQR)	30	(25–37)
Range (year)	20–69	
**Race/Ethnicity^a^**		
Asian [NILM, ASC-US, LSIL, HSIL]	32 [8, 8, 10, 6]	(3.6)
Black [NILM, ASC-US, LSIL, HSIL]	106 [28, 29, 35, 14]	(12.0)
White [NILM, ASC-US, LSIL, HSIL]	333 [84, 96, 82, 71]	(37.7)
Unknown [NILM, ASC-US, LSIL, HSIL]	407 [115, 102, 99, 91]	(46.1)
Missing [NILM, ASC-US, LSIL, HSIL]	5 [2, 0, 3, 0]	(0.6)
**Cytological^b^**		
Total LBC samples collected	883	(100)
LBC samples missing clinical data^c^	5	(0.6)
NILM	2	(0.2)
ASCUS	0	(0)
LSIL	3	(0.4)
HSIL	0	(0)
**LBC samples included**	883	(100)
NILM	237	(27)
ASCUS	235	(26)
LSIL	229	(26)
HSIL	181	(21)
HSIL/SCC	1	(0.1)
**Source^d^**		
Cervical	875	(99.1)
Vaginal	3	(0.3)
Unspecified	5	(0.6)
**Diagnostic category^d^**		
Normal	237	(27)
Abnormal	646	(73)
***Cellular DNA concentration^d,e,f^***		
Total LBC samples [Median (ng/uL) (IQR)]	95.6	(54.3–168.4)
NILM [Median (ng/uL) (IQR)]	91.6	(54.2–147.3)
ASCUS [Median (ng/uL) (IQR)]	81.1	(34.9–147.3)
LSIL [Median (ng/uL) (IQR)]	119.2	(67.5–202.0)
HSIL [Median (ng/uL) (IQR)]	104.0	(54.8–178.2)

*ASC-US, atypical squamous cells of undetermined significance; IQR, interquartile range; LBC, Liquid-based Cytology; LSIL, low-grade squamous intraepithelial lesion; HSIL, high-grade squamous intraepithelial lesion; NILM, negative for intraepithelial lesion and malignancy; SCC, squamous cell carcinoma. ^a^Age and Race/Ethnicity of subjects (N = 883) are based on the demographic data of included samples. The number of samples by race/ethnicity and cytological diagnosis are placed in brackets. The distribution of the 5 categories of race/ethnicity (inclusive of Unknown and Missing data) across the 4 cytological grades were not different (χ^2^, p = 0.354). ^b^Cytopathology results are ascribed to the specimens collected on day of study enrollment. ^c^Exclusion criteria included: low cell pellet volume (<200 μL). ^d^Data based on all included samples (N = 883). Unspecified source (cervix or vagina). ^e^Concentration of total cellular DNA per sample after semi-automated DNA extraction. ^f^Comparison of DNA concentrations between NILM, ASC-US, LSIL, and HSIL sample groups showed slightly lower concentration for NILM vs. LSIL, ASC-US vs. LSIL, and ASC-US vs. HSIL (p < 0.05) by the Wilcoxon rank-sum test. ^h^Values are N (%) unless otherwise denoted.*

**FIGURE 2:**
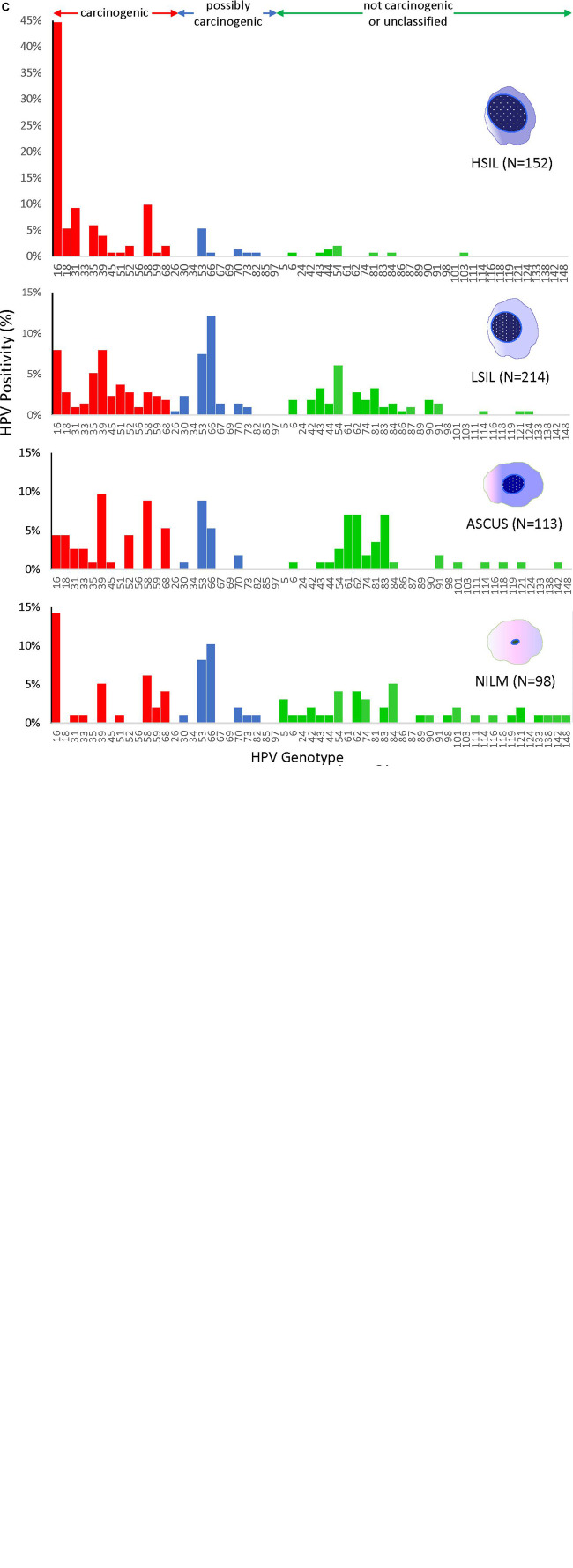
HPV prevalence and genotype distribution found in 4 cytological grades. **(A)** HPV DNA positivity rate for 883 samples as determined by PCR amplification and gel electrophoresis. The positive rates for NILM and ASCUS were over 50%, whereas the rates were significantly higher for LSIL and HSIL at ∼80–90% (top) (*p < 0.05, chi-square test). **(B)** Distribution of HPV-positive rates stratified by type-specific carcinogenic potential for PCR-positive/sequenced samples (*n* = 640). Progression of cytological grade from NILM to HSIL correlated with a significant uptrend in carcinogenic HPV-types and a downtrend in possibly and not carcinogenic/unclassified HPV-types (*p < 0.05, chi-square trend test). Samples with poor or noisy sequence quality unidentifiable by BLAST also decreased with worsening cytological grade (*p < 0.05, chi-square trend test). **(C)** HPV genotype distribution of 577 cytology samples as determined by PCR/Sanger sequencing according to cytological diagnoses. The remaining 63 HPV-positive samples could not be genotyped due to poor sequence quality and/or overlapping sequences of mixed infections. The proportion of carcinogenic HPV genotypes (red bars) increased coincidently with cytological grade (*p < 0.05, chi-square trend test). In contrast, the possibly and not carcinogenic/unclassified HPV-types (blue and green bars, respectively) significantly diminished (*p < 0.05, chi-square trend test). Simultaneously, species richness diminished from NILM to HSIL (38 to 23 genotypes, respectively) while HPV-16 surged in 68/152 (45%) HSIL samples. ASC-US, atypical squamous cells of undetermined significance; CARC, carcinogenic HPV; HSIL, high- grade squamous intraepithelial lesion; LSIL, low-grade squamous intraepithelial lesion; NA, not available/identifiable by BLAST; NILM, negative for intraepithelial lesion or malignancy; NOT CARC, not carcinogenic; NS, not significant; POSS CARC, possibly carcinogenic Stars, *p* < 0.01.

HPV prevalence determined by PCR and gel electrophoresis increased significantly with worsening grade: NILM (53%), ASC-US (58%), LSIL (97%), and HSIL (86%) (Figure 2A). Similarly, the proportion of carcinogenic HPV genotypes in PCR-positive sequenced samples (*n* = 640) increased coincidently with cytological grade: NILM (27%), ASC-US (37%), LSIL (41%), and HSIL (82%) (Figure 2B). Conversely, a significant downtrend was found for HPV genotypes in possibly, not carcinogenic/unclassified HPV-types, and HPV-types that were unidentifiable by BLAST (*p* < 0.05, chi-square trend test).

The HPV genotype distribution of 577 sequenced cytology samples spanned the continuum of IARC-defined carcinogenic potentials (Figure 2C). The remaining 63 HPV PCR-positive samples could not be genotyped by BLAST due to uninterpretable (poor or noisy) sequencing results. Species richness diminished remarkably with progression of cytopathology from NILM to HSIL (38 to 23 genotypes, respectively). HPV16 surged and dominated the HSIL viral community in 68/152 (45%) samples. Meanwhile, the 12 other carcinogenic and a few possibly carcinogenic and not carcinogenic/unclassified genotypes dwindled but persisted in HSIL samples.

**FIGURE 3:**
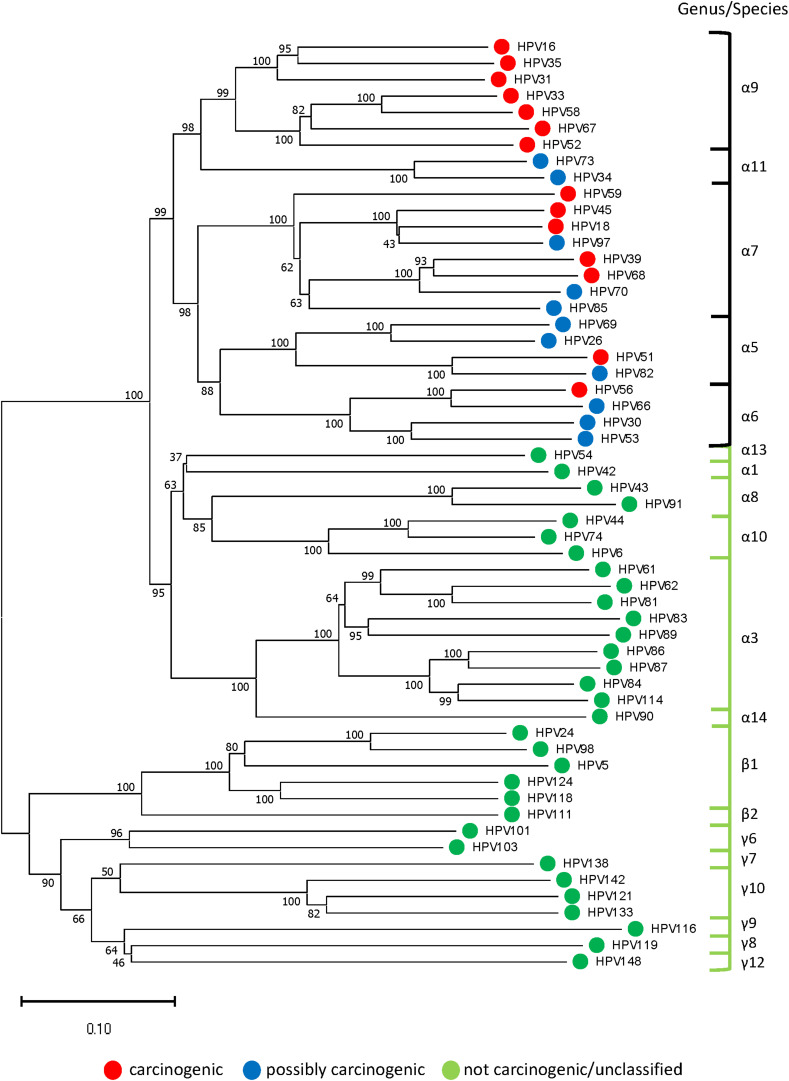
Representative phylogenetic tree of HPV genotypes identified in the clinical samples. Neighbor-Joining tree of 57 HPV genotypes (one from each genotype) revealed two distinct clades in the *alpha* genera: “high-risk” containing carcinogenic and possibly carcinogenic types [black bracket] and “low-risk” containing probably not carcinogenic or not classifiable types [green bracket]. The *beta and gamma* genera formed another clade composed of commensal and unclassified genotypes. With HPV-16 at the pinnacle of HPV carcinogenic potential, genetic divergence from this point correlated with decreased carcinogenic risk (phenotype) and grade of cytopathology. The evolutionary history was inferred using the Neighbor-Joining method after concatenating 57 aligned, E6, E7, and L1 reference nucleotide sequences from Papillomavirus Episteme by MUSCLE (Edgar, 2004). The optimal tree with the sum of branch length = 9.99536245 is shown. The percentage of replicate trees in which the associated taxa clustered together in the bootstrap test (1000 replicates) are shown next to the branches (Felsenstein, 1985). The tree is drawn to scale, with branch lengths in the same units as those of the evolutionary distances used to infer the phylogenetic tree. The evolutionary distances were computed using the Maximum Composite Likelihood method (Tamura et al., 2004) and are in the units of the number of base substitutions per site. All ambiguous positions were removed for each sequence pair (pairwise deletion option). There were a total of 2850 positions in the final dataset. Evolutionary analyses were conducted in MEGA X (Kumar et al., 2018).

A representative Neighbor-Joining tree constructed from reference sequences of 57 HPV genotypes (one for each genotype identified in the 577 sequenced samples) is presented in Figure 3. The concatenated sequences grouped likewise to the conventional L1-based and joined E7/E1/E2/L2/L1-based phylogenetic trees) (International Agency for Research on Cancer [IARC], 2012; Schiffman et al., 2009). The tree revealed an inverse relationship between genetic distance from HPV-16 (highest carcinogenic potential) and carcinogenic risk which corresponded to the prevalent HPV genotypes found among the four grades of cytology as shown in Figure 2C.

### Promoter Hypermethylation of ADCY8, CDH8, and ZNF582 Correlates With Cytological Grade

**FIGURE 4:**
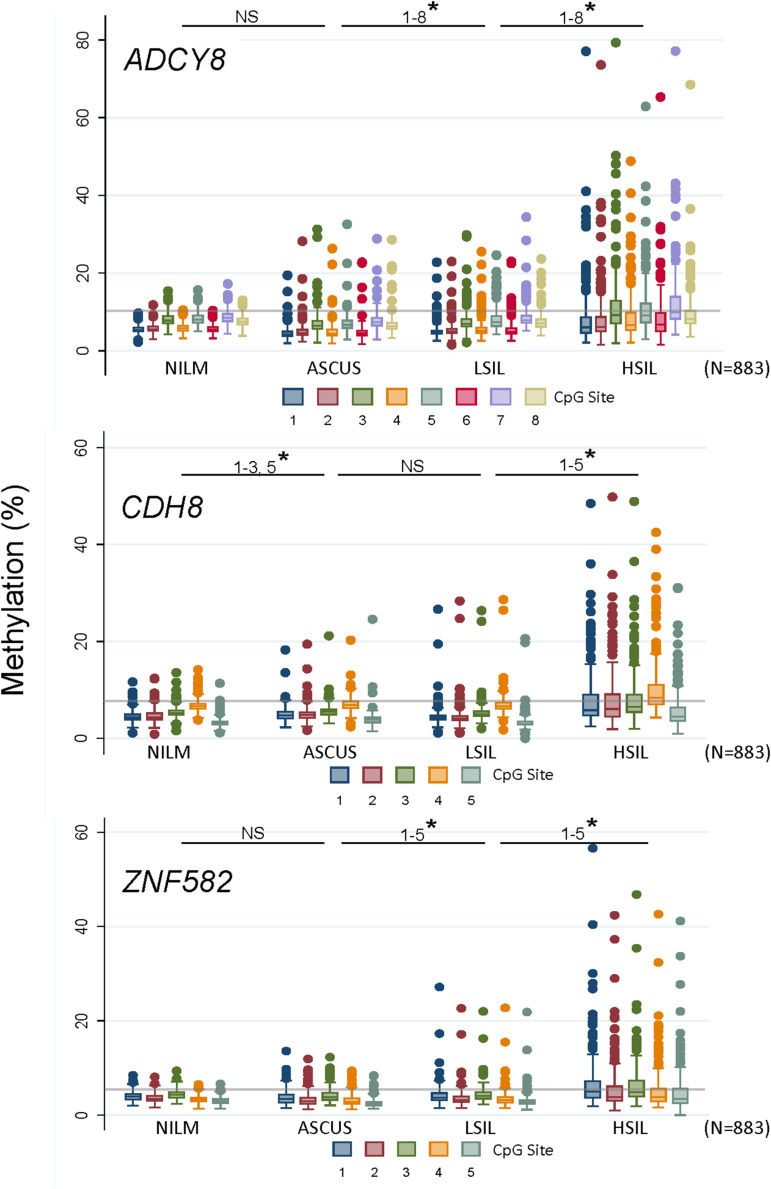
Loci-specific promoter methylation differences and trends among cervical cytological grades. Methylation (%) of total genomic DNA in 4 grades of cervical cytology i.e., NILM (*n* = 237), ASC-US (*n* = 235), LSIL (*n* = 229), and HSIL (*n* = 182) was compared by CpG sites among 3 genes (*ADCY8*, *CDH8*, and *ZNF582*). Pairwise comparisons of methylation for each CpG site between cytological grades (NILM vs. ASC-US, ASC-US vs. LSIL, and LSIL vs. HSIL) revealed significantly higher levels at multiple sites as noted by a star (* *p* < 0.05 by the Wilcoxon rank-sum test). Methylation levels for all CpG sites increased coincidently with cytological grade for *ADCY8*, *CDH8*, and *ZNF582* by Spearman’s *r*_*s*_ (*p* < 0.05, with Bonferroni adjustment) except for *ADCY8* CpG sites 1–5. The methylation reference line (gray) for each assay denotes the median of the 95th percentile values for each CpG site within an assay derived from NILM (HPV-negative) samples, i.e., *ADCY8* (10.11%), *CDH8* (7.61%), and *ZNF582* (5.22%). ASC-US, atypical squamous cells of undetermined significance; HSIL, high-grade squamous intraepithelial lesion; LSIL, low-grade squamous intraepithelial lesion; NILM, negative for intraepithelial lesion or malignancy; NS, not statistically significant.

Methylation levels for all CpG sites increased coincidently with worsening cytological grade for *ADCY8, CDH8*, and *ZNF582* except for *ADCY8* at CpG sites 1-5 (Spearman rank, *p* < 0.05). Pairwise comparisons of methylation for each CpG site between cytological grades (NILM vs. ASC-US, ASC-US vs. LSIL, and LSIL vs. HSIL) revealed significantly higher levels at multiple sites for the worse grade denoted by a star in Figure 4 (^∗^*p* < 0.05 by the Wilcoxon rank-sum test). The upper limit of normal for CpG-methylation levels summarized as the median of the 95th percentile of each CpG site per assay were: *ADCY8* (10.11%), *CDH8* (7.61%), and *ZNF582* (5.22%). Positive control sample #503 bore methylation levels 2-fold and 3-fold that of NILM cytology for *ADCY8* and *CDH8/ZNF582* assays, respectively (Supplementary Figure 3). CpG assay results analyzed on the PyroMark Q48 were also compared to Q96 data from our previous study (Shen-Gunther et al., 2016). This revealed a slightly higher CpG-methylation level for each site on the Q48 indicative of improved analytical sensitivity (Supplementary Figure 4).

### ADCY8, CDH8, and ZNF582 CpG-Markers Contribute to HPV as a Predictor of Cytological Grade

**FIGURE 5:**
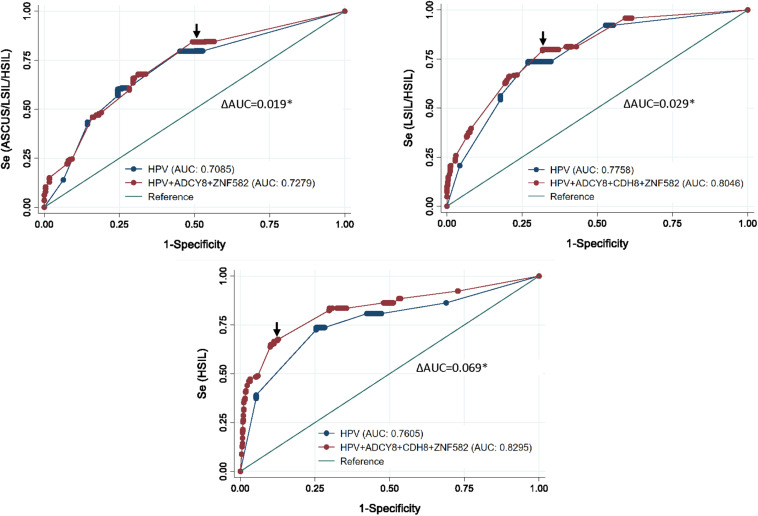
Receiver operating characteristic (ROC) curve analysis after multivariable logistic regression for three logit models. Top left, the ROC curve revealed the best predictors to differentiate between NILM and ASCUS/LSIL/HSIL as HPV carcinogenicity, *ZNF582*_1st CpG site, and *ADCY8*_5th CpG site. Top right, for differentiating between NILM/ASCUS and LSIL/HSIL cytology, the best multivariate predictor was the combination of HPV carcinogenicity, *ZNF582*_1st CpG site, *CDH8*_4th CpG site, and *ADCY8*_5th CpG site. Bottom, for differentiating between NILM/ASCUS/LSIL and HSIL cytology, the best multivariate predictor was the combination of HPV carcinogenicity, *ZNF582*_1st CpG site, *CDH8*_4th CpG site, and *ADCY8*_6th CpG site. All three multivariable models were better predictors of the specified outcome than HPV carcinogenicity alone (delta AUC*, *p* < 0.05, chi-square test). AUC, Area under the receiver operator curve (AUC); se, sensitivity; cut-off points (arrows).

ROC curve analysis after multivariable logistic regression for three logit models are presented in Figure 5. The best predictors for differentiating NILM from ASC-US/LSIL/HSIL was HPV carcinogenicity, *ZNF582*_1st CpG site, and *ADCY8*_5th CpG site (Supplementary Table 1). The best multivariable predictor for differentiating between NILM/ASC-US and LSIL/HSIL cytology was the combination of HPV carcinogenicity, *ZNF582*_1st CpG site, *CDH8*_4th CpG site, and *ADCY8*_5th CpG site (Supplementary Table 2). Additionally, the combination of HPV carcinogenicity, *ZNF582*_1st CpG site and *CDH8*_4th CpG site, and *ADCY8*_6th CpG site is the best multivariable predictor for differentiating between NILM/ASC-US/LSIL and HSIL cytology (Supplementary Table 3). All three multivariable models were better predictors of the specified outcome than HPV carcinogenicity alone (delta AUC^∗^, *p* < 0.05, chi-square test). Overall, the number of missing observations for HPV genotype 63/883 (7.1%) and site-specific CpG-methylation ranging from 1 to 9/883 (≤ 1%) were low prior to imputation and predictive modeling. The variables with counts of missing observations are listed in Supplementary Table 4.

### HPV With Host CpG-Markers Outperforms HPV as a Classifier of Cervical Cytology

**FIGURE 6:**
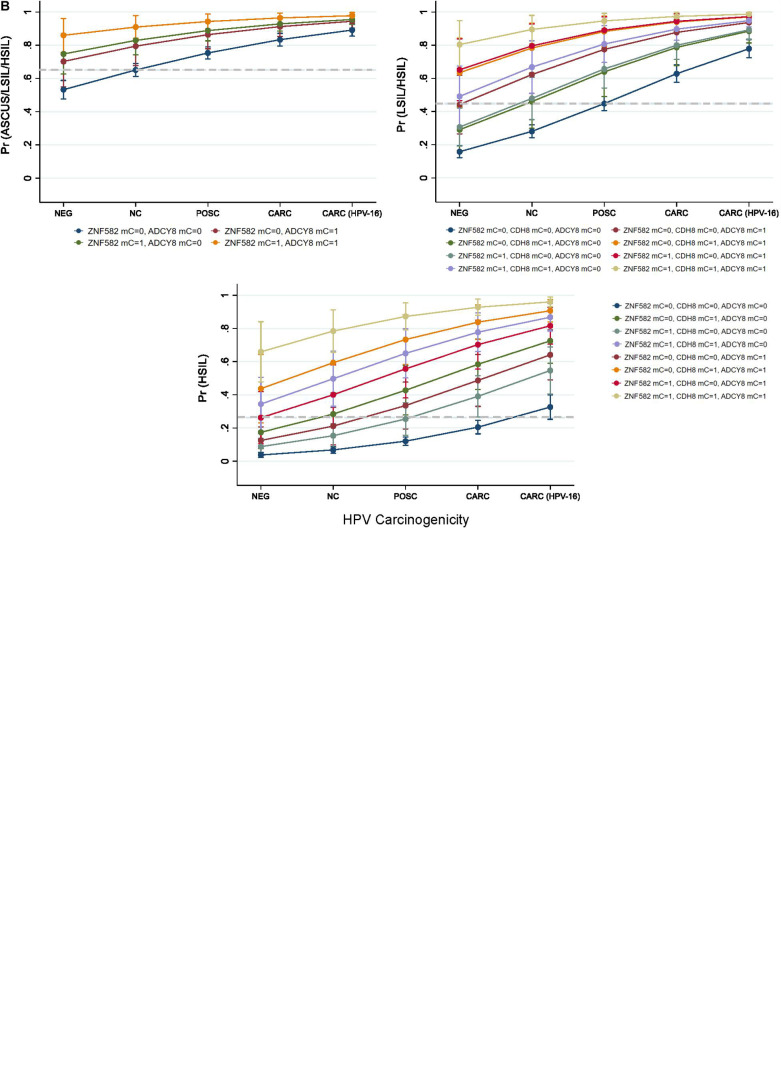
Predicted probabilities plot of binarized cytological outcomes using HPV carcinogenicity as a singular or integrated predictor variable. **(A)** HPV carcinogenicity as a one-dimensional predictor of 3 sequentially binarized cytological outcomes (NILM vs. ASC-US/LSIL/HSIL, NILM/ASCUS vs. LSIL/HSIL, and NILM/ASC-US/LSIL vs. HSIL) is shown with respective cut-off values of ≥0.680, 0.5222, and 0.3321 (dashed lines) as determined by Youden’s index. **(B)** HPV carcinogenicity and host loci-specific methylation as predictors of cytological outcome. Top left, comparison of predicted probabilities for abnormal cytology (NILM vs. ASC-US/LSIL/HSIL) by HPV carcinogenicity and binarized *ZNF582* and *ADCY8* methylation status. Top right, comparison of predicted probabilities for NILM/ASC-US vs. LSIL/HSIL permuted by binarized methylation values of *ADCY8, CDH8*, and *ZNF582* at the CpG sites noted in the text. Bottom, comparison of predicted probabilities for <HSIL vs. HSIL permuted by binarized methylation values of *ADCY8, CDH8*, and *ZNF582* at the CpG sites noted in the text. The cut-off values for predicting a positive binarized cytological outcomes (NILM vs. ASC-US/LSIL/HSIL, NILM/ASC-US vs. LSIL/HSIL, and NILM/ASC-US/LSIL vs. HSIL) were ≥0.6503, 0.4533, and 0.2645 (dashed lines) as determined by Youden’s index. Definitions: For loci-specific CpG methylation levels (%), the 95th percentile value for each CpG derived from HPV-negative. NILM cytology was used as the cut-off for normal methylation (coded as 0); >95th percentile was deemed hypermethylated (coded as 1). AUC, area under the curve; mC, 5-methylcytosine at CpG sites; mC = 0, unmethylated cytosine; mC = 1, methylated cytosine; Pr, probability; ROC, Receiver operating characteristic; Se, sensitivity.

Predicted probabilities plots of binarized cytological outcomes using HPV carcinogenicity as a singular or integrated predictor variable are shown in Figure 6. HPV carcinogenicity as a one-dimensional predictor of 3 sequentially binarized cytological outcomes (NILM vs. ASC-US/LSIL/HSIL, NILM/ASC-US vs. LSIL/HSIL, and NILM/ASC-US/LSIL vs. HSIL) are shown with respective cut-off values of ≥0.680, 0.5222, and 0.3321 as determined by Youden’s index (Figure 6A; Youden, 1950). HPV-16 was distinct from the other carcinogenic HPVs in predicting the cytological outcome of interest.

HPV carcinogenicity and host loci-specific methylation as predictors of cytological outcome are shown in Figure 6B. The 3 subgraphs illustrate the escalating probability for the cytological outcome of interest coincident with HPV carcinogenicity and increased number of methylated genes. For example, the probability for HSIL escalated with HPV carcinogenicity and increased counts of methylated genes reaching the pinnacle of 96% at HPV-16 jointly with 3 methylated genes. Furthermore, the contribution of each methylated gene to the probability of the outcome of interest are different singularly or in combination with others as shown by non-overlapping lines (Figure 6B). The cut-off values for predicting positive binarized cytological outcomes (NILM vs. ASCUS/LSIL/HSIL, NILM/ASCUS vs. LSIL/HSIL, and NILM/ASCUS/LSIL vs. HSIL) were ≥0.6503, 0.4533, and 0.2645 as determined by Youden’s index (Youden, 1950).

The diagnostic performance characteristics of the three logit models were presented in Supplementary Tables 5–7. Specifically, models 1 and 2 inclusive of methylation markers showed greater sensitivity (83% and 79%, respectively) and a lower negative likelihood ratio compared to HPV alone. This implies that HPV with methylation markers performed better at predicting absence of disease (more true negative results) and in differentiating normal (NILM) from abnormal (>NILM) in model 1 as well as NILM/ASC-US from LSIL/HSIL in model 2. In contradistinction, model 3 performed better at predicting presence of disease (more true positive results) with greater specificity (88%) and had a higher positive likelihood ratio of 5.55. In other words, a positive result was ∼5 times more likely to occur in a patient with HSIL than one with < HSIL.

## Discussion

This study aimed to validate the predictive model of a multiparametric biomarker panel based on HPV genotype and host epigenetic modifications for cervical cytopathology. The expanded results of the current study confirmed our previous findings, which determined that HPV carcinogenicity is positively correlated with aberrant DNA methylation in the promoters of *ADCY8*, *CDH8*, and *ZNF582* in addition to the four cytological grades (Shen-Gunther et al., 2016). In this study, the addition of ASC-US samples revealed a distinct and intermediate HPV genotype prevalence pattern between NILM and LSIL. Similarly, the promoter methylation levels of ASC-US were found between that of NILM and LSIL. Overall, the multivariate biomarker panel improved the prediction and classification of cytological grade over the univariate HPV carcinogenicity.

The HPV genotype patterns among the cytological categories revealed a loss of species diversity and gain of dominance by carcinogenic types as cytology progressed from NILM to HSIL. Our PCR results showed a doubling in HPV positivity rate from NILM/ASC-US to LSIL/HSIL samples (>80–90%). PCR-sequencing revealed a steep rise in the proportion of carcinogenic genotypes as cytology progressed to HSIL with the 3 topmost being HPV-16 (45%), HPV-58 (10%), and HPV-31 (9%). In contrast, the possibly carcinogenic and non-carcinogenic HPV genotypes declined precipitously in prevalence and diversity indicative of extinction of the “weaker competitors” within the dynamic virus-host ecosystem. This phenomenon, demonstrated previously by our deep sequencing study, may be explained by the principles of competitive exclusion followed by clonal expansion of HPV-infected transformed cells (Ueda et al., 2003, 2008; Shen-Gunther et al., 2017). The competitive advantage of carcinogenic, particularly HPV-16, versus non-carcinogenic HPV is derived from the significantly higher binding affinity of viral E6 and E7 oncoproteins to host cellular proteins p53 and pRB, respectively, leading to subsequent degradation, genomic instability, and carcinogenesis (Chemes et al., 2010; Martinez-Zapien et al., 2016; Tomita et al., 2020; ðukić et al., 2020). Furthermore, the distinctive cellular binding motifs of E6 and E7 oncoproteins, which correlate with tissue and host specificity, is believed to be the result of virus-host adaptive evolution over millions of years (Chemes et al., 2012; Chen et al., 2018; Suarez and Trave, 2018). Evolutionary analysis of 57 representative genotypes confirmed the inverse relationship between genetic distance from HPV-16 and carcinogenic potential. As a corollary, the type-specific carcinogenic risk was reflected in the severity of cytopathology.

Cellular epigenetic analyses confirmed our previous findings of a positive correlation between promoter methylation of *ADCY8*, *CDH8*, and *ZNF582* and cytological grade (Shen-Gunther et al., 2016). The curvilinear upward trend in quantitated methylation levels for all 3 genes was validated on the high-resolution PyroMark Q48 instrument. Our results are consistent with E6 and E7 oncoprotein induced promotion of *de novo* and/or maintenance DNA methyltransferase (DNMT1, DNMT3A, and DNMT3B) (Burgers et al., 2007; Au Yeung et al., 2010; Durzynska et al., 2017). The loci-specific methylation profiles from the Q48 mirrored that of Q96 from our previous study (Shen-Gunther et al., 2016). However, CpG-site specific median methylation levels were consistently slightly higher for Q48 which ranged from 1 to 8%. We ascribe this finding to the advanced chemistry and improved algorithms employed in the Q48 resulting in reduced background and augmented sensitivity in sequencing reactions (Qiagen, 2020). High-resolution PSQ offered a technological advantage in this study by exposing subtle differences in methylation levels between gene specific CpG sites and identifying the CpG site that contributed most to our predictive models. Along this line, Lioznova and coauthors dubbed unique single CpG methylation sites as “CpG traffic lights” which were found to correlate more often with gene expression and repression than an averaged promoter methylation statistic (Medvedeva et al., 2014; Lioznova et al., 2019).

Model refitting was performed due to sample size expansion, ASC-US sample inclusion, and PSQ technological advancement in this cohort. The combination of HPV carcinogenicity and methylation markers remained significant predictors of cytological outcome after multivariable logistic regression. The addition of methylation status improved the sensitivity and/or specificity for the binarized outcome of interest for all 3 models. The predicted probability for the outcome escalated in a stepwise fashion as HPV carcinogenicity reached its pinnacle, i.e., HPV-16. This finding is consistent with a longitudinal study where HPV-16, in comparison to other carcinogenic HPV types, was found to convey a uniquely elevated risk for severe cervical intraepithelial neoplasia (CIN3 +) (Demarco et al., 2020). Furthermore, as the number of methylated genes increased, the probability for the outcome increased. Whereas slight differences were noted between specific genes. Together, infection with the most carcinogenic HPV and maximal loci methylation predicted the greatest probability for high-grade cytology.

Discriminatory performance of the 3 multi-parametric models demonstrated greater accuracy than the univariate HPV carcinogenicity. Specifically, models 1 and 2 inclusive of methylation markers showed greater sensitivity and a lower negative likelihood ratio. This implies that HPV with methylation markers performed better at predicting absence of disease (more true negative results). Conversely, model 3 performed better at predicting presence of disease (more true positive results) with greater specificity and a higher positive likelihood ratio of 5.55. Likelihood ratios, akin to signal-to-noise ratios, are robust measures of diagnostic accuracy that are independent of disease prevalence (Deeks and Altman, 2004; Lang and Secic, 2006). This statistic allows for generalizability and comparison beyond the scope of this study. Finally, our predictive models and classification schemes designed for sequential use will enable the allocation of an unknown sample to the appropriate cytological category (Long and Freese, 2014).

Published literature on promoter methylation of *ADCY8, CDH8*, and *ZNF582* has expanded recently and hypermethylation of one or more of these loci have been found in cancers of the breast, oropharynx, esophagus, and anus (Tang et al., 2019; Ekanayake Weeramange et al., 2020; Sigin et al., 2020; van der Zee et al., 2020). These reports not only support the validity of these epigenetic modifications as cancer biomarkers but inform a broader application beyond cervical cancer. First, differential methylation of *ADCY8* was identified as one of three most informative biomarkers in luminal B breast cancer from a cohort of Russian women. Methylation status of these markers were predictive of response to neoadjuvant chemotherapy before surgery which may be applicable as a clinical test to guide therapy (Sigin et al., 2020). Second, *CDH8* promoter hypermethylation has been documented in four head and neck cancer studies (Ekanayake Weeramange et al., 2020). One such study used PSQ for validation of epigenetic alteration in 70 oropharyngeal squamous cell carcinomas. HPV-positive, in contrast to HPV-negative tumors, was found to be significantly correlated with hypermethylation and prognosis in this Japanese cohort (Nakagawa et al., 2017).

*ZNF582* is the best studied of our 3 methylation markers. *ZNF582* promoter hypermethylation has been confirmed in multiple of studies of cervical precancerous lesions, and invasive adeno- and squamous carcinomas (Huang et al., 2012; Chang et al., 2014; Lai et al., 2014; Lin et al., 2014; Liang et al., 2020). In fact, the analysis of *ZNF582* by methylation-specific quantitative PCR is being commercialized as an *in vitro* diagnostic test (Beltrán-García et al., 2019). Hypermethylated *ZNF582* and *PAX1* genes have also been found in mouth rise samples applicable to the detection of oral dysplasia and cancer (Cheng et al., 2018). In Esophageal Squamous Cell Carcinoma (ESCC), aberrant hypermethylation of *ZNF582* and *PAX1* have been demonstrated using quantitative methylation-specific PCR with levels at 21% versus 0% for tumor and peri-tumor normal tissues, respectively (Huang et al., 2017). Another study of esophageal cancer found significantly higher methylation levels by PSQ in cancerous than adjacent non-cancerous and normal tissues, respectively: 31%, 11%, and 15% (Tang et al., 2019). Finally, in a Netherlands study of 345 anal intraepithelial neoplasia (AIN grades 1-3) and invasive carcinoma samples, *ZNF582* methylation levels escalated with increased disease severity. Among the markers studied, *ZNF582* was the most accurate for detecting AIN grade 3 with immense potential as a clinical biomarker (van der Zee et al., 2020). Taken together, *ADCY8, CDH8*, and *ZNF582* promoter methylation are promising predictive and prognostic biomarkers for multiple tumor types crossing geographic and racial boundaries that undeniably merits further validation.

The strength of this study lies in the inclusion of ASC-US samples and sample size expansion for all cytological categories. This led to increased precision and power of distinction between the four grade-specific HPV communities and methylation levels for model validation. Additionally, high resolution PSQ played a critical role in pinpointing the gene specific CpG that contributed the most to the predictive models, as well as, exposing the subtle differences between CpG sites. PSQ has also been proven as a superior method to methylation-specific PCR for prognostication of survival outcomes (Johannessen et al., 2018). We acknowledge that our study has limitations in that two remaining, uncommon cytological categories with potentially different risk profiles, i.e., ASC-H and AGUS were not included (Nayar et al., 2020). To fill this gap, ASC-H and AGUS samples have been collected for our ongoing large-scale study (>3,000 samples), which is intended to complete our investigation and understanding of molecular evolution within a dynamic virus-host ecosystem.

## Conclusion

Our expanded findings validated the multivariable prediction model developed for cytological classification. The sequencing-based “Molecular Pap smear” outperformed the singular HPV carcinogenicity in predicting four grades of cervical cytology. Additional host epigenetic markers that evolved with disease progression contributed to the overall classification accuracy.

## Author’s Note

This paper has undergone PAO review at Brooke Army Medical Center and was cleared for publication. The view(s) expressed herein are those of the authors and do not reflect the official policy or position of Brooke Army Medical Center, the United States Army Medical Department, the United States Army Office of the Surgeon General, the Department of the Army, the Department of the Air Force, or the Department of Defense or the United States Government.

Presented virtually at the 33rd International Papillomavirus Conference (IPVC 2020) on July 23, 2020.

## Data Availability Statement

Primary sequence data will be deposited in the NCBI GenBank upon patent issuance.

## Ethics Statement

This study was approved by the institutional review board of Brooke Army Medical Center, Fort Sam Houston, Texas.

## Author Contributions

JS-G conceived and designed the study and analyzed and interpreted the data. JS-G, QX, WS, and HA participated in the acquisition of data and wrote the manuscript. All authors read and approved the final manuscript.

## Conflict of Interest

The United States Army Medical Research and Development Command has filed a patent application on the invention described herein. The inventor is JS-G. No potential conflicts of interest were disclosed by the other authors.

## Footnotes

1 http://pave.niaid.nih.gov

## Supplementary Material

Supplementary Figure 1 Representative Hpv genomes and target regions used for Hpv Dna detection by Pcr amplification.

Supplementary Figure 2 Representative electropherograms and gel images of target amplicons from *Adcy8, Cdh8*, and *Znf582* promoter regions for downstream pyrosequencing.

Supplementary Figure 3 Pyrograms of *Adcy8*, *Cdh8*, and *Znf582* for sample # 503 with a diagnosis of Hsil/Squamous cell carcinoma on cervical cytology.

Supplementary Figure 4 Comparison of *Adcy8, Cdh8*, and *Znf582* CpG assay results analyzed on the PyroMark Q48 and Q96 platforms.

Supplementary Table 1 Logistic regression analysis of Hpv and Hpv + *Adcy8* + *Znf582* for predicting abnormal (Asc-Us/Lsil/Hsil) cytology.

Supplementary Table 2 Logistic regression analysis of Hpv and Hpv + 3-gene methylation markers for predicting abnormal (Lsil/Hsil) cytology.

Supplementary Table 3 Logistic regression analysis of Hpv and Hpv + 3-gene methylation markers for predicting for Hsil cytology.

Supplementary Table 4 Predictor variables used for logistic regression with missing observations.

Supplementary Table 5 Diagnostic performance of Hpv vs. Hpv + *Adcy8* + *Znf582* for predicting abnormal (Asc-Us/Lsil/Hsil) cytology.

Supplementary Table 6 Diagnostic performance of Hpv vs. Hpv + 3-gene methylation markers for predicting Lsil/Hsil cytology.

Supplementary Table 7 Diagnostic performance of Hpv vs. Hpv + 3-gene methylation markers for predicting Hsil cytology.

## Abbreviations

*ADCY8*: adenylate cyclase 8
ASC-US: atypical squamous cells of undetermined significance
AUC: Area under the receiver operator curve
CARC: carcinogenic HPV
*CDH8*, cadherin 8: type 2
CIN: cervical intraepithelial neoplasia
chr: chromosome
gDNA: genomic DNA
HPV: Human Papillomavirus
HSIL: high-grade squamous intraepithelial lesion
IARC: International Agency for Research on Cancer
LSIL: low-grade squamous intraepithelial lesion
NA: not available/identifiable by BLAST
NILM: negative for intraepithelial lesion or malignancy
NOT CARC: not carcinogenic
NS: not significant
Pap: Papanicolaou smear
POSS CARC: possibly carcinogenic
PSQ: pyrosequencing
Q48: PyroMark Q48 platform
Q96: PyroMark Q96 platform
ROC: receiver operating characteristic
SCC: squamous cell carcinoma
*ZNF582*: zinc finger protein 582.
