# Supplementary material for: Molecular Pap Smear: Validation of HPV Genotype and Host Methylation Profiles of ADCY8, CDH8, and ZNF582 as a Predictor of Cervical Cytopathology
Source: Front Microbiol. 2020 Oct 15;11:595902. doi: 10.3389/fmicb.2020.595902 (PMC7593258; doi:10.3389/fmicb.2020.595902)
Supplement: Supplementary Figure 1 — Representative Hpv genomes and target regions used for Hpv Dna detection by Pcr amplification. [file Data_Sheet_1.PDF]

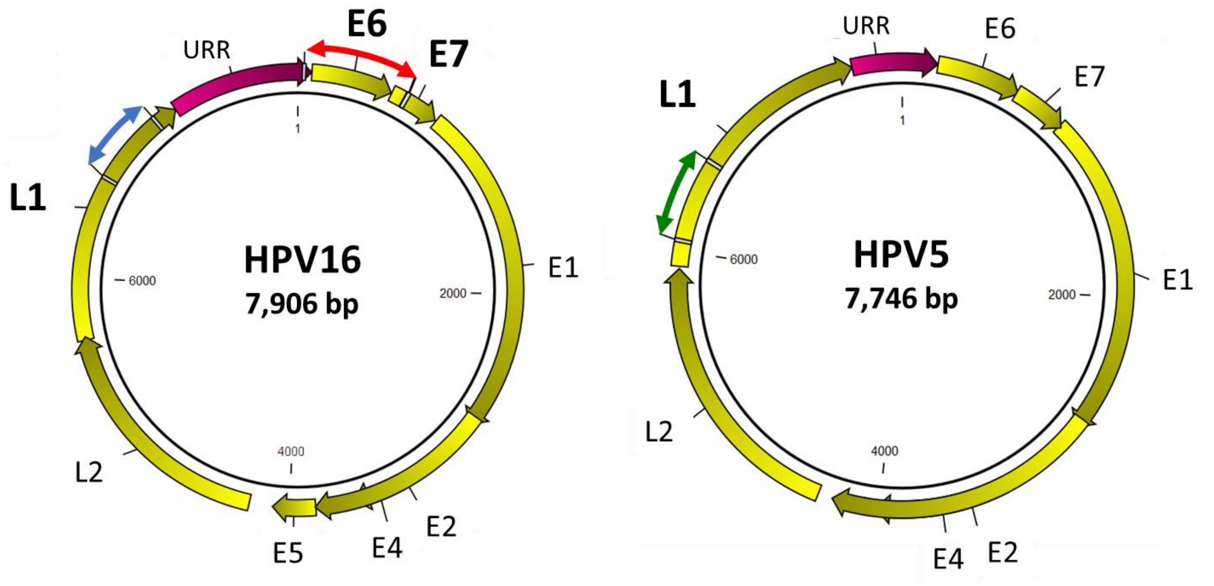

Supplementary Figure 1. Representative HPV genomes and target regions used for HPV DNA detection by PCR amplification. The HPV genome is a ~8,000 base pair, double-stranded, circular DNA. The prototypical genome encodes 6 early genes (E1, E2, E4, E5, E6 and E7) and 2 late genes (L1 and L2). The MY09/11 consensus primers designed for broad spectrum coverage of mucosal HPV genotypes target the late (L1) capsid gene (↔) between nucleotide positions 6584/7035 as shown on the HPV-16 genome. Consensus primers (GP-E6-3F/GP-E7-5B/GP-E7-6B) target two early (E6 and E7) genes that encode oncoproteins. The E6/E7 fragment (↔) is located between nucleotide positions 28/658 on the HPV-16 genome. The FAP59/64 consensus primers originally designed for broad spectrum coverage of cutaneous HPV genotypes (which may also detect mucosal genotypes) target the late (L1) capsid gene (↔) between nucleotide positions 6047/6527 as shown on the HPV-5 genome. The HPV genomes were constructed using CLC Genomics Workbench 20.0.4 (Qiagen) based on the genetic information of HPV-16 from the *alpha* genus (GenBank ID: K02718) and HPV-5 from the *beta* genus (GenBank ID: M17463). bp, base pair; URR, upstream regulatory region
