# Supplementary material for: Molecular Pap Smear: Validation of HPV Genotype and Host Methylation Profiles of ADCY8, CDH8, and ZNF582 as a Predictor of Cervical Cytopathology
Source: Front Microbiol. 2020 Oct 15;11:595902. doi: 10.3389/fmicb.2020.595902 (PMC7593258; doi:10.3389/fmicb.2020.595902)
Supplement: Supplementary Figure 2 — Representative electropherograms and gel images of target amplicons from Adcy8, Cdh8, and Znf582 promoter regions for downstream pyrosequencing. [file Data_Sheet_2.PDF]

A

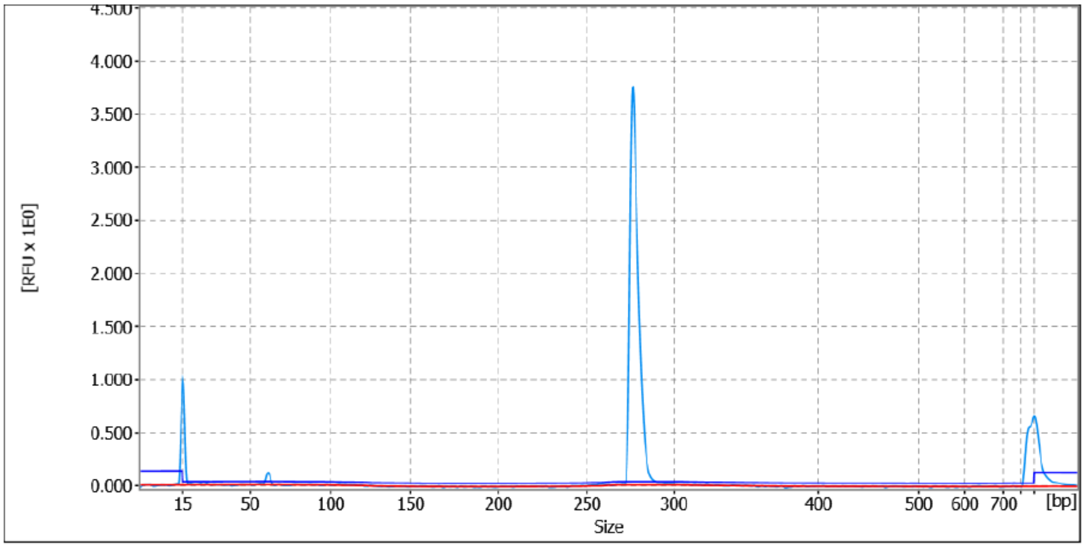

4.500
4.000
3.500
3.000
2.500
2.000
1.500
1.000
0.500
0.000
[RFU x 1E0]
15
50
100
150
200
250
300
400
500
600
700
[bp]
Size

Figure: 57

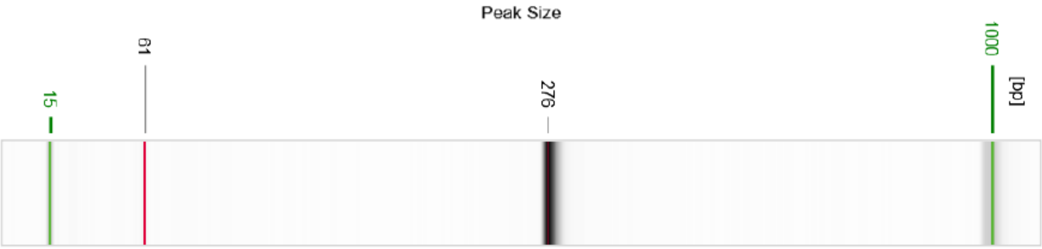

Peak Size
15
61
276
1000
[bp]

B

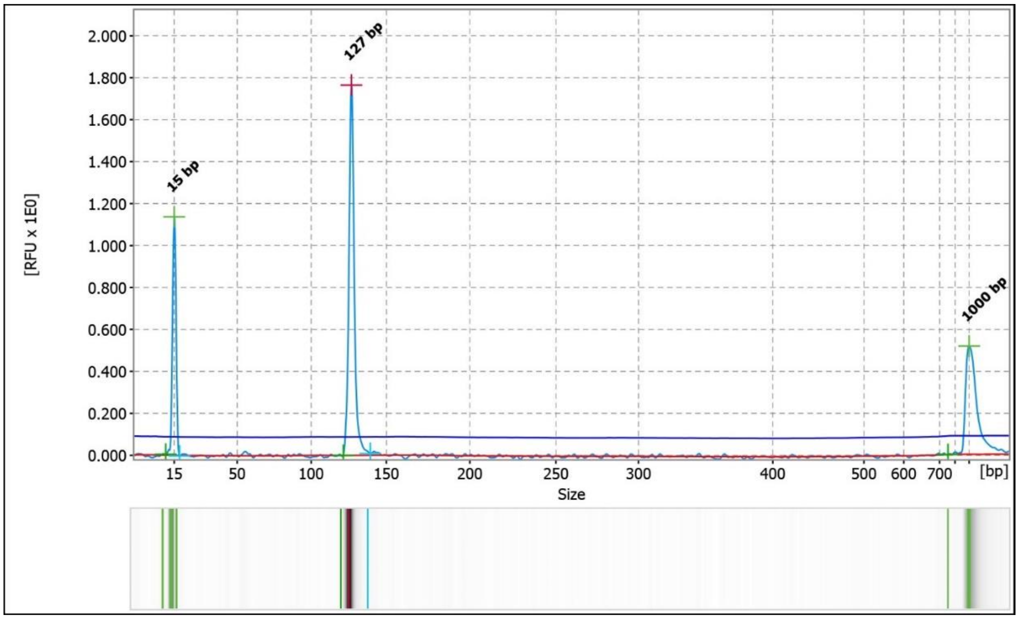

C

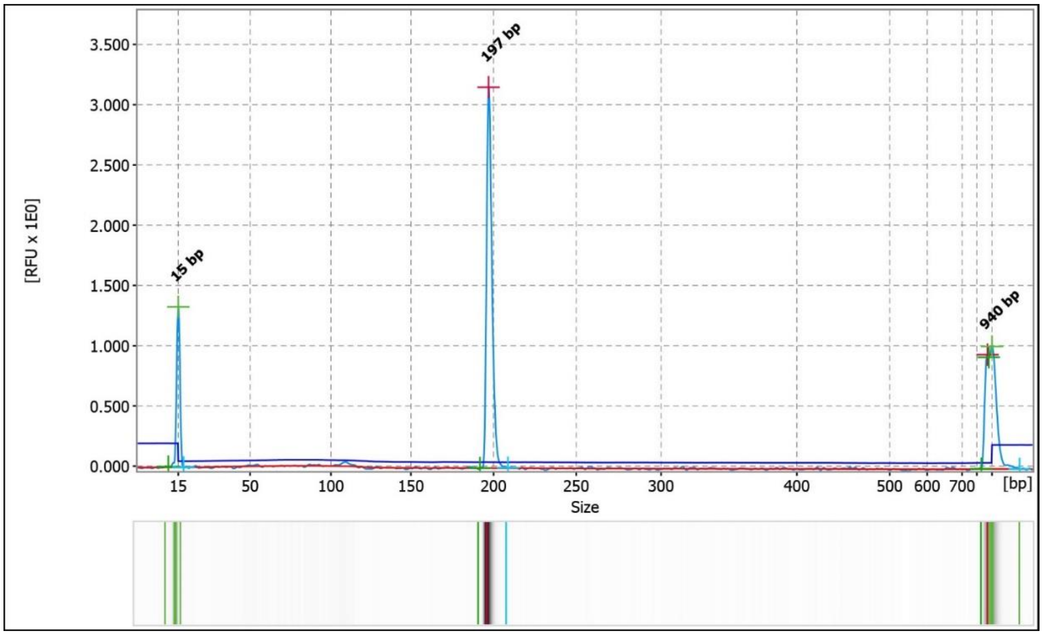

Supplementary Figure 2. PCR amplification of host genomic DNA targeting *ADCY8*, *CDH8* and *ZNF582* promoter regions. Representative electropherogram and gel image of target amplicon detected by high-resolution capillary electrophoresis for: (**A**) *ADCY8* (276 bp), (**B**) *CDH8* (127 bp), and (**C**) *ZNF582* (197 bp). The amplicons were used for downstream pyrosequencing. Images were derived from sample #503 (positive control) with a cytological diagnosis of HSIL/SCCA. bp, base pair; HSIL, high-grade squamous intraepithelial lesion; SCCA, squamous cell carcinoma.
