# Supplementary material for: Molecular Pap Smear: Validation of HPV Genotype and Host Methylation Profiles of ADCY8, CDH8, and ZNF582 as a Predictor of Cervical Cytopathology
Source: Front Microbiol. 2020 Oct 15;11:595902. doi: 10.3389/fmicb.2020.595902 (PMC7593258; doi:10.3389/fmicb.2020.595902)
Supplement: Supplementary Figure 3 — Pyrograms of Adcy8, Cdh8, and Znf582 for sample # 503 with a diagnosis of Hsil/Squamous cell carcinoma on cervical cytology. [file Data_Sheet_3.PDF]

A

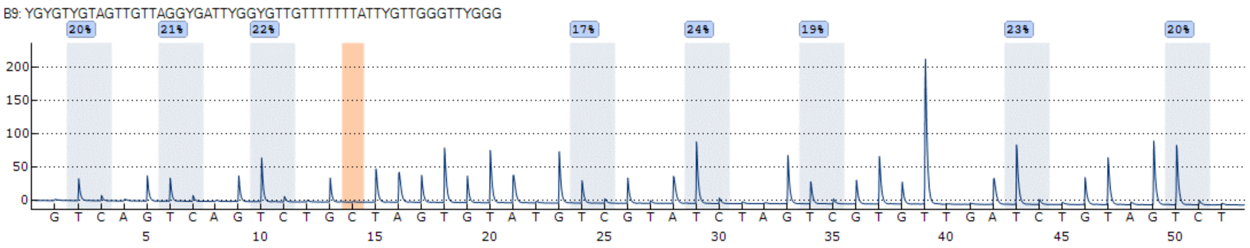


B

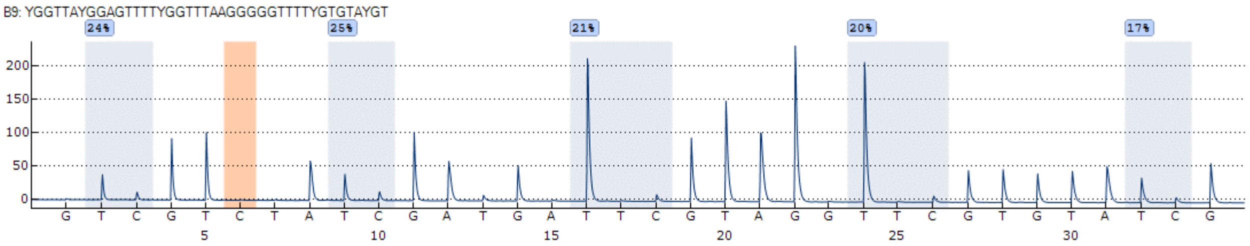


C

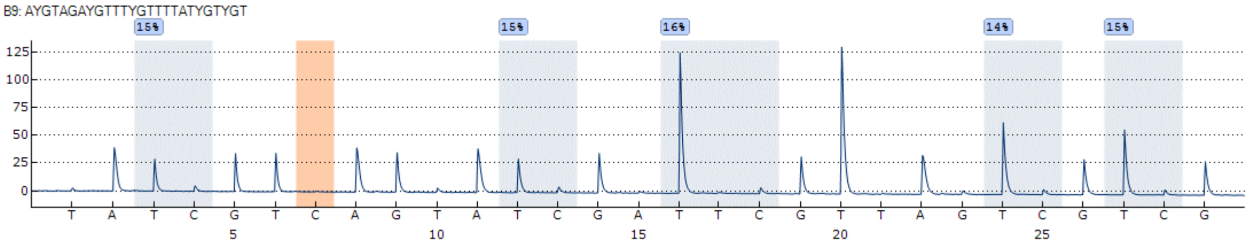


**Supplementary Figure 3.** CpG-methylation of promoter regions analyzed by pyrosequencing on the PyroMark Q48 platform. Pyrograms were derived from sample #503 (positive control) with a cytological diagnosis of HSIL/SCC. Hypermethylated CpG levels are shown for: (**A**) *ADCY8* assay (8 CpG sites), (**B**) *CDH8* assay (5 CpG sites), and (**C**) *ZNF582* assay (5 CpG sites). The methylation levels for *ADCY8* and *CDH8/ZNF582* were 2-fold and 3-fold that of NILM cytology, respectively. The blue-gray shaded columns indicate the CpG dinucleotides assayed with the percentage of methylated cytosine residues at the top. The 95 percentile of CpG-methylation (%) for NILM cytology by respective sites were: *ADCY8* CpG sites 1-8 (8.35, 7.99, 11.88, 9.40, 11.88, 8.65, 12.75, 10.81); *CDH8* sites 1-5 (7.04, 7.94, 7.61, 9.71, 5.12), and *ZNF582* CpG sites 1-5 (5.92, 5.22, 6.31, 4.54, 4.52). Pyrogram x- and y-axis represent the sequence to analyze and light-intensity peaks, respectively. HSIL, high-grade squamous intraepithelial lesion; SCCA, squamous cell carcinoma.
