# Supplementary material for: Molecular Pap Smear: Validation of HPV Genotype and Host Methylation Profiles of ADCY8, CDH8, and ZNF582 as a Predictor of Cervical Cytopathology
Source: Front Microbiol. 2020 Oct 15;11:595902. doi: 10.3389/fmicb.2020.595902 (PMC7593258; doi:10.3389/fmicb.2020.595902)
Supplement: Supplementary Figure 4 — Comparison of Adcy8, Cdh8, and Znf582 CpG assay results analyzed on the PyroMark Q48 and Q96 platforms. [file Data_Sheet_4.PDF]

A

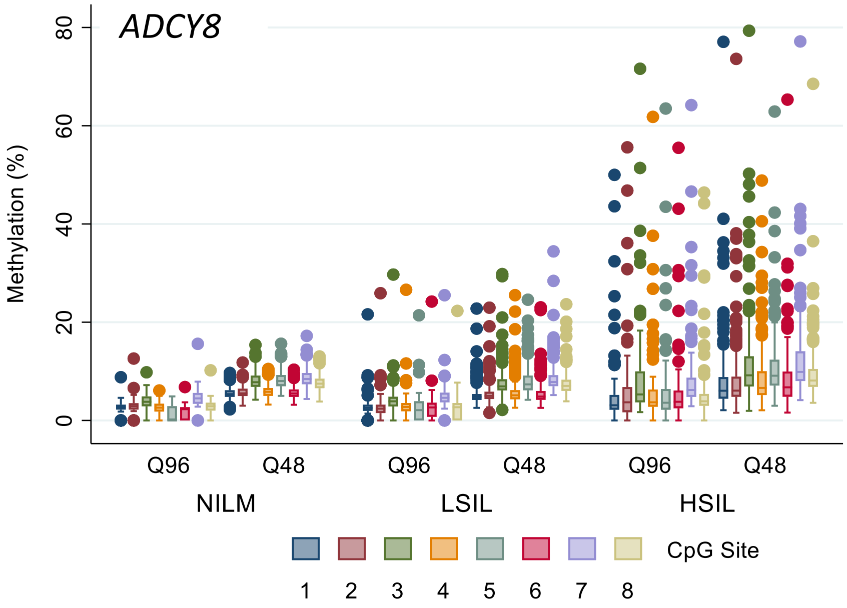

ADCY8
Methylation (%)
0
20
40
60
80
Q96
Q48
Q96
Q48
Q96
Q48
NILM
LSIL
HSIL
CpG Site
1
2
3
4
5
6
7
8

B

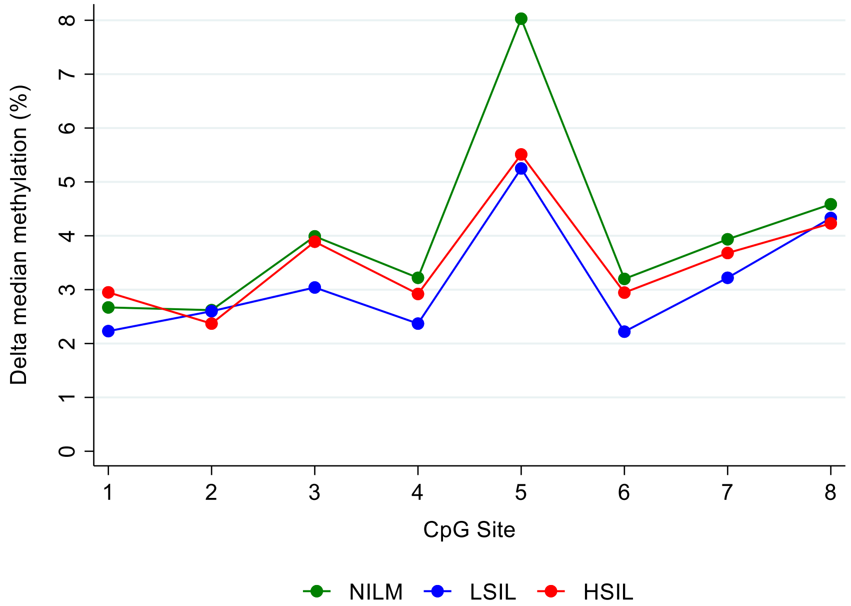

Delta median methylation (%)
0
1
2
3
4
5
6
7
8
1
2
3
4
5
6
7
8
CpG Site
NILM
LSIL
HSIL

C

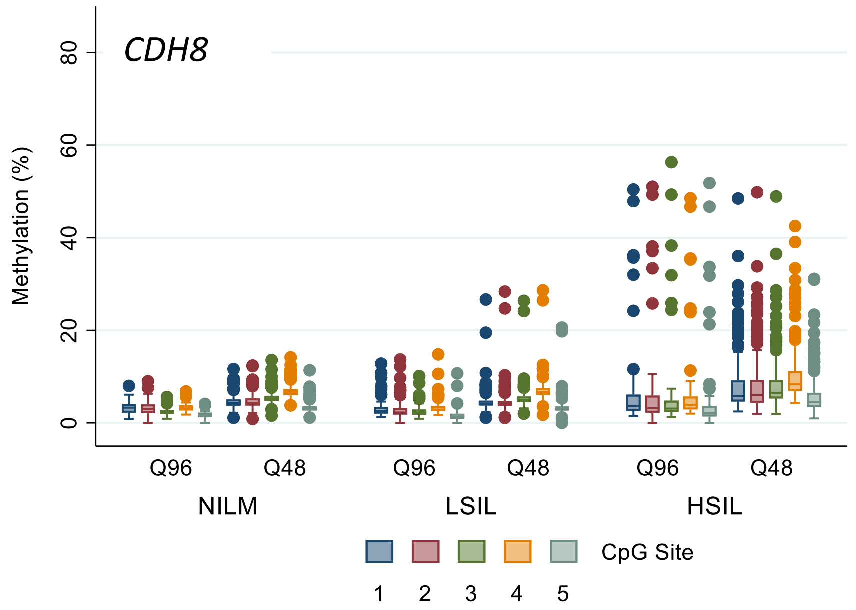
CDH8
Methylation (%)
80
60
40
20
0
Q96
Q48
Q96
Q48
Q96
Q48
NILM
LSIL
HSIL
CpG Site
1
2
3
4
5

D

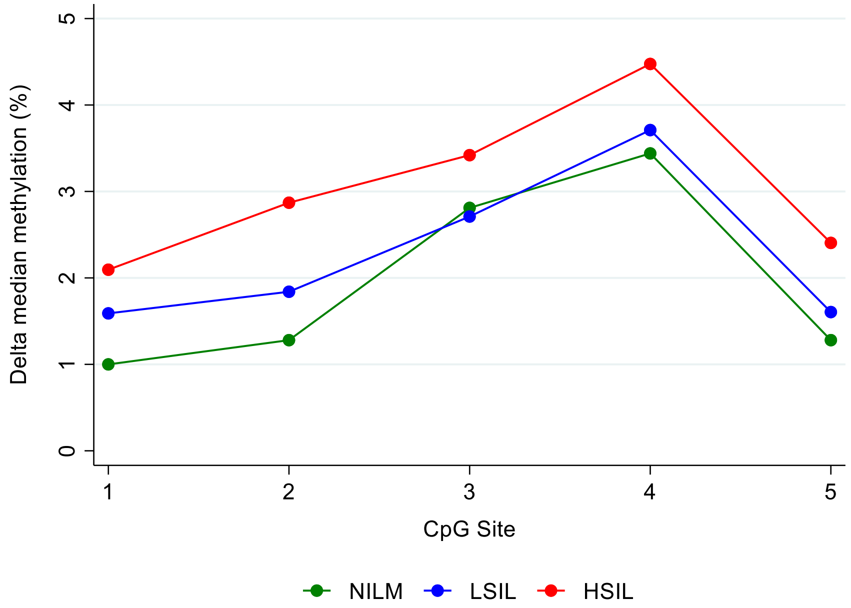
Delta median methylation (%)
5
4
3
2
1
0
1
2
3
4
5
CpG Site
NILM
LSIL
HSIL

E

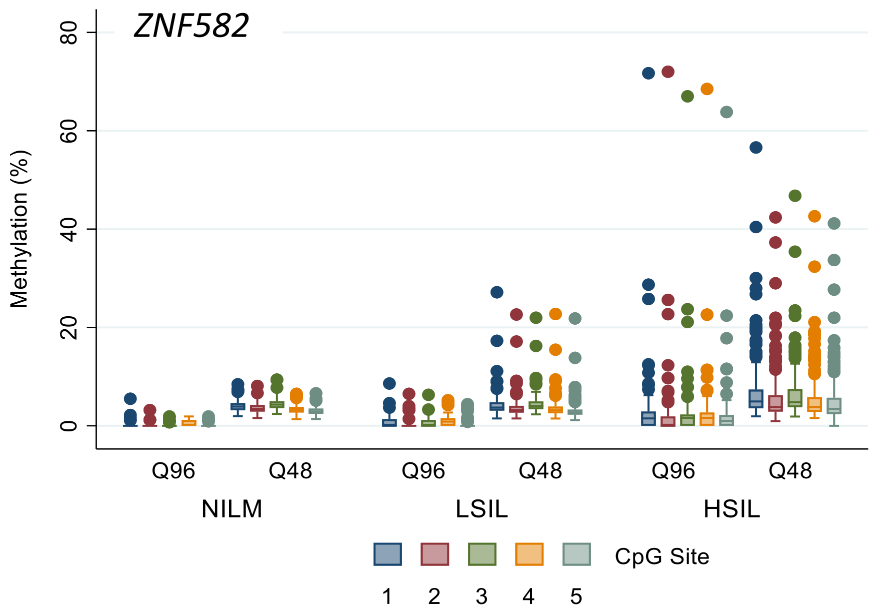

F

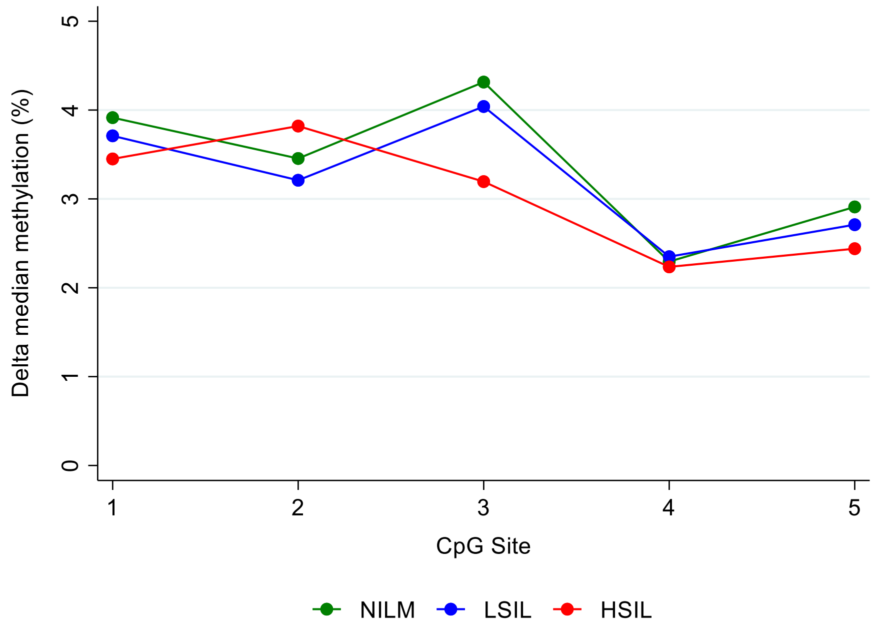

Supplementary Figure 4. Comparison of CpG assay results analyzed on the PyroMark Q48 and Q96 platforms. The Q48 with improved accuracy consistently detected higher methylation levels for all CpG sites within NILM, LSIL and HSIL samples for *ADCY8* (**A**), *CDH8* (**C**) and *ZNF582* (**E**). For each CpG site, the difference between median methylation levels (Q48 minus Q96) was consistent across cytological grades for the 3 assays (**B, D, and F**) indicative of test repeatability. In contrast, the difference between CpG sites was varied among assays. NILM, LSIL and HSIL samples (*n*) tested on the Q48 and Q96 were (237, 229, and 181) and (33, 70, and 67), respectively.
