# Supplementary material for: Molecular Pap Smear: Validation of HPV Genotype and Host Methylation Profiles of ADCY8, CDH8, and ZNF582 as a Predictor of Cervical Cytopathology
Source: Front Microbiol. 2020 Oct 15;11:595902. doi: 10.3389/fmicb.2020.595902 (PMC7593258; doi:10.3389/fmicb.2020.595902)
Supplement: Supplementary Table 1 — Logistic regression analysis of Hpv and Hpv + Adcy8 + Znf582 for predicting abnormal (Asc-Us/Lsil/Hsil) cytology. [file Data_Sheet_5.PDF]

Supplementary Table 1. Logistic regression analysis of HPV and HPV + *ADCY8* + *ZNF582* for predicting abnormal (ASCUS/LSIL/HSIL) cytology

Variable	Coefficient (β)	SE	t	P>t	95% CI		
			Univariable model[a]				
HPV[b]	0.551	0.067	8.20	0.000	0.419	to	0.683
constant	0.214	0.112	1.90	0.057	-0.007	to	0.434
			Multivariable model[a]				
HPV[b]	0.521	0.069	7.55	0.000	0.385	to	0.656
ADCY8[c]	0.803	0.405	1.99	0.047	0.010	to	1.596
ZNF582[d]	0.931	0.322	2.89	0.004	0.300	to	1.563
constant	0.113	0.117	0.97	0.332	-0.116	to	0.342

HPV, human papillomavirus; HSIL, high-grade squamous intraepithelial lesion; LSIL, low-grade squamous intraepithelial lesion; SE, standard error; t, t-score.

[a]Logistic regression performed after multiple imputation for missing data (m = 20 imputations used).

[b]The HPV genotype identified in each sample was coded accordingly: HPV undetected (0), not classifiable (1), possibly carcinogenic (2), carcinogenic (3), and carcinogenic HPV-16 (4).

[c]The quantified promoter methylation value (%) of *ADCY8* gene at CpG-position 5 of each sample was binarized accordingly: ≤11.88 (0), >11.88 (1).

[d]The quantified promoter methylation value (%) of *ZNF582* gene at CpG-position 1 of each sample was binarized accordingly: ≤5.92 (0), > 5.92 (1).
