# Supplementary material for: Molecular Pap Smear: Validation of HPV Genotype and Host Methylation Profiles of ADCY8, CDH8, and ZNF582 as a Predictor of Cervical Cytopathology
Source: Front Microbiol. 2020 Oct 15;11:595902. doi: 10.3389/fmicb.2020.595902 (PMC7593258; doi:10.3389/fmicb.2020.595902)
Supplement: Supplementary Table 2 — Logistic regression analysis of Hpv and Hpv + 3-gene methylation markers for predicting abnormal (Lsil/Hsil) cytology. [file Data_Sheet_6.PDF]

Supplementary Table 2. Logistic regression analysis of HPV and HPV + *ADCY8* + *CDH8*+ *ZNF582* for predicting abnormal (LSIL/HSIL) cytology

Variable	Coefficient (β)	SE	t	P>t	95% CI		
			Univariable model[a]				
HPV[b]	0.766	0.061	12.56	0.000	0.647	to	0.886
constant	-1.451	0.129	-11.25	0.000	-1.704	to	-1.198
			Multivariable model[a]				
HPV[b]	0.734	0.064	11.52	0.000	0.609	to	0.859
ADCY8[c]	1.490	0.361	4.13	0.000	0.782	to	2.197
CDH8[d]	0.712	0.336	2.12	0.034	0.053	to	1.372
ZNF582[e]	0.809	0.272	2.98	0.003	0.277	to	1.342
constant	-1.663	0.139	-11.98	0.000	-1.936	to	-1.391

HPV, human papillomavirus; HSIL, high-grade squamous intraepithelial lesion; LSIL, low-grade squamous intraepithelial lesion; SE, standard error; t, t-score.

[a]Logistic regression performed after multiple imputation for missing data (m = 20 imputations used).

[b]The HPV genotype identified in each sample was coded accordingly: HPV undetected (0), not classifiable (1), possibly carcinogenic (2), carcinogenic (3), and carcinogenic HPV-16 (4).

[c]The quantified promoter methylation value (%) of *ADCY8* gene at CpG-position 5 of each sample was binarized accordingly: ≤11.88 (0), >11.88 (1).

[d]The quantified promoter methylation value (%) of *CDH8* gene at CpG-position 4 of each sample was binarized accordingly: ≤9.71 (0), > 9.71 (1).

[e]The quantified promoter methylation value (%) of *ZNF582* gene at CpG-position 1 of each sample was binarized accordingly: ≤5.92 (0), > 5.92 (1).
