# Supplementary material for: Molecular Pap Smear: Validation of HPV Genotype and Host Methylation Profiles of ADCY8, CDH8, and ZNF582 as a Predictor of Cervical Cytopathology
Source: Front Microbiol. 2020 Oct 15;11:595902. doi: 10.3389/fmicb.2020.595902 (PMC7593258; doi:10.3389/fmicb.2020.595902)
Supplement: Supplementary Table 3 — Logistic regression analysis of Hpv and Hpv + 3-gene methylation markers for predicting for Hsil cytology. [file Data_Sheet_7.PDF]

Supplementary Table 3. Logistic regression analysis of HPV and HPV + *ADCY8* + *CDH8*+ *ZNF582* for predicting abnormal (HSIL) cytology

Variable	Coefficient (β)	SE	t	P>t	95% CI		
			Univariable model[a]				
HPV[b]	0.743	0.072	10.380	0.000	0.602	to	0.883
constant	-2.926	0.201	-14.570	0.000	-3.320	to	-2.533
			Multivariable model[a]				
HPV[b]	0.638	0.077	8.290	0.000	0.487	to	0.789
ADCY8[c]	1.351	0.321	4.210	0.000	0.722	to	1.980
CDH8[d]	1.614	0.314	5.140	0.000	0.999	to	2.229
ZNF582[e]	0.892	0.270	3.300	0.001	0.362	to	1.422
constant	-3.266	0.220	-14.840	0.000	-3.697	to	-2.834

HPV, human papillomavirus; HSIL, high-grade squamous intraepithelial lesion; LSIL, low-grade squamous intraepithelial lesion; SE, standard error; t, t-score.

[a]Logistic regression performed after multiple imputation for missing data (m = 20 imputations used).

[b]The HPV genotype identified in each sample was coded accordingly: HPV undetected (0), not classifiable (1), possibly carcinogenic (2), carcinogenic (3), and carcinogenic HPV-16 (4).

[c]The quantified promoter methylation value (%) of *ADCY8* gene at CpG-position 6 of each sample was binarized accordingly: ≤8.65 (0), >8.65 (1).

[d]The quantified promoter methylation value (%) of *CDH8* gene at CpG-position 4 of each sample was binarized accordingly: ≤9.71 (0), >9.71 (1).

[e]The quantified promoter methylation value (%) of *ZNF582* gene at CpG-position 1 of each sample was binarized accordingly: ≤5.92 (0), >5.92 (1).
