# Supplementary material for: Molecular Pap Smear: Validation of HPV Genotype and Host Methylation Profiles of ADCY8, CDH8, and ZNF582 as a Predictor of Cervical Cytopathology
Source: Front Microbiol. 2020 Oct 15;11:595902. doi: 10.3389/fmicb.2020.595902 (PMC7593258; doi:10.3389/fmicb.2020.595902)
Supplement: Supplementary Table 4 — Predictor variables used for logistic regression with missing observations. [file Data_Sheet_8.PDF]

Supplementary Table 4. Variables with missing observations for multiple imputation and logistic regression analysis

	Missing Observations[a]		Non-Missing Observations		Total
Variable[b]	(*n*)	(%)	(*n*)	(%)	(*N*)
HPV genotype	63	7.1	820	92.9	883
ADCY8_CpG1	4	0.5	879	99.5	883
ADCY8_CpG2	4	0.5	879	99.5	883
ADCY8_CpG3	4	0.5	879	99.5	883
ADCY8_CpG4	5	0.6	878	99.4	883
ADCY8_CpG5	5	0.6	878	99.4	883
ADCY8_CpG6	5	0.6	878	99.4	883
ADCY8_CpG7	6	0.7	877	99.3	883
ADCY8_CpG8	6	0.7	877	99.3	883
CDH8_CpG1	0	0	883	100.0	883
CDH8_CpG2	0	0	883	100.0	883
CDH8_CpG3	0	0	883	100.0	883
CDH8_CpG4	0	0	883	100.0	883
CDH8_CpG5	1	0.1	882	99.9	883
ZNF582_CpG1	5	0.6	878	99.4	883
ZNF582_CpG2	7	0.8	876	99.2	883
ZNF582_CpG3	8	0.9	875	99.1	883
ZNF582_CpG4	8	0.9	875	99.1	883
ZNF582_CpG5	9	1.0	874	99.0	883

ADCY8, adenylate cyclase 8; *CDH8*, cadherin 8; CpG*n*, CpG *n* site; *ZNF582*, zinc finger protein 582.

[a]Percentage of missing observations was calculated as follows: *n* missing observations/883 total samples. Logistic regression was performed after multiple imputation for missing observations (m = 20 imputations used).

[b]Missing observations for HPV genotype were due to uninterpretable (poor or noisy) sequencing results. Missing observations for loci-specific CpG-methylation levels were due to suboptimal pyrosequencing results categorized as "check" or "failed" after auto-analysis by the software.
