# Supplementary material for: Molecular Pap Smear: Validation of HPV Genotype and Host Methylation Profiles of ADCY8, CDH8, and ZNF582 as a Predictor of Cervical Cytopathology
Source: Front Microbiol. 2020 Oct 15;11:595902. doi: 10.3389/fmicb.2020.595902 (PMC7593258; doi:10.3389/fmicb.2020.595902)
Supplement: Supplementary Table 5 — Diagnostic performance of Hpv vs. Hpv + Adcy8 + Znf582 for predicting abnormal (Asc-Us/Lsil/Hsil) cytology. [file Data_Sheet_9.PDF]

Supplementary Table 5. Diagnostic performance of HPV vs. HPV + *ADCY8* + *ZNF582* for predicting abnormal (ASCUS/LSIL/HSIL) cytology

Parameters	Biomarkers	
	HPV	HPV + *ADCY8* + *ZNF582*
Disease (cytological)[a]		
Present (ASCUS/LSIL/HSIL)	646 (73)	646 (73)
Absent (NILM)	237 (27)	237 (27)
Total	883 (100)	883 (100)
Diagnostic performance[b, c]		
Sensitivity	509/646 (79)	537/646 (83)
Specificity	126/237 (53)	115/237 (49)
PPV	509/620 (82)	537/659 (81)
NPV	126/263 (48)	115/224 (51)
False-positive	111/237 (47)	122/237 (51)
False-negative	137/646 (21)	109/646 (17)
LR + test (TP/FP)	1.68	1.61
LR - test (FN/TN)	0.40	0.35
Accuracy, %	635/883 (72)	652/883 (74)

FN, false negative; FP, false positive; HPV, human papillomavirus; HSIL, high-grade squamous intraepithelial lesion; LR, likelihood ratio; LSIL, low-grade squamous intraepithelial lesion; NILM, negative for intraepithelial lesion/malignancy; TN, true negative; TP, true positive; ZNF, *ZNF582* gene.

[a]The disease state was defined by the cytological diagnosis of the specimen at study entry. It served as the reference standard for the clinical performance of the molecular tests i.e., HPV and HPV + *ADCY8 + ZNF582*.

[b]The positive outcome or "classification threshold" probabilities used for classification of outcomes for HPV and HPV + *ADCY8* + *ZNF582* were ≥0.680 and 0.650, respectively.

[c]Values are n/N (%) unless denoted otherwise.
