# Supplementary material for: Molecular Pap Smear: Validation of HPV Genotype and Host Methylation Profiles of ADCY8, CDH8, and ZNF582 as a Predictor of Cervical Cytopathology
Source: Front Microbiol. 2020 Oct 15;11:595902. doi: 10.3389/fmicb.2020.595902 (PMC7593258; doi:10.3389/fmicb.2020.595902)
Supplement: Supplementary Table 7 — Diagnostic performance of Hpv vs. Hpv + 3-gene methylation markers for predicting Hsil cytology. [file Data_Sheet_11.PDF]

Supplementary Table 7. Diagnostic performance of HPV vs. HPV + *ADCY8* + *CDH8*+ *ZNF582* for predicting abnormal (HSIL) cytology

	Biomarkers	
Parameters	HPV	HPV + *ADCY8* + *CDH8* + *ZNF582*
Disease (cytological)[a]		
Present (HSIL)	182 (21)	182 (21)
Absent (NILM/ASCUS/LSIL)	701 (79)	701 (79)
Total	883 (100)	883 (100)
Diagnostic performance[b, c]		
Sensitivity	133/182 (73)	121/182 (67)
Specificity	510/701 (73)	617//701 (88)
PPV	133/324 (41)	121/205 (59)
NPV	510/559 (91)	617/678 (91)
False-positive	191/701 (27)	84/701 (12)
False-negative	49/182 (27)	61/182 (34)
LR + test (TP/FP)	2.68	5.55
LR - test (FN/TN)	0.37	0.38
Accuracy, %	643/883 (73)	738/883 (84)

ADCY, *ADCY8* gene; CDH, *CDH8* gene; FN, false negative; FP, false positive; HPV, human papillomavirus; HSIL, high-grade squamous intraepithelial lesion; LR, likelihood ratio; LSIL, low-grade squamous intraepithelial lesion; NILM, negative for intraepithelial lesion/malignancy; TN, true negative; TP, true positive; ZNF, *ZNF582* gene.

[a]The disease state was defined by the cytological diagnosis of the specimen at study entry. It served as the reference standard for the clinical performance of the molecular tests i.e., HPV and HPV + 3-gene methylation markers.

[b]The positive outcome or “classification threshold” probabilities used for classification of outcomes for HPV and HPV + 3-gene methylation markers were ≥0.332 and 0.265, respectively.

[c]Values are n/N (%) unless denoted otherwise.
